# Inhibition of Microsomal Prostaglandin E2 Synthase Reduces Collagen Deposition in Melanoma Tumors and May Improve Immunotherapy Efficacy by Reducing T-cell Exhaustion

**DOI:** 10.1158/2767-9764.CRC-23-0210

**Published:** 2023-07-31

**Authors:** Yasunari Fukuda, Sun-Hee Kim, Matias A. Bustos, Sung-Nam Cho, Jason Roszik, Jared K. Burks, Hong Kim, Dave S.B. Hoon, Elizabeth A. Grimm, Suhendan Ekmekcioglu

**Affiliations:** 1 Department of Melanoma Medical Oncology, The University of Texas MD Anderson Cancer Center, Houston, Texas.; 2 Department of Translational Molecular Medicine and Genome Sequencing, Saint John's Cancer Institute, Providence Saint John's Health Center, Santa Monica, California.; 3 Department of Genomic Medicine, The University of Texas MD Anderson Cancer Center, Houston, Texas.; 4 Department of Leukemia, The University of Texas MD Anderson Cancer Center, Houston, Texas.; 5 Department of Bioinformatics and Computational Biology, The University of Texas MD Anderson Cancer Center, Houston, Texas.; 6 MD Anderson Cancer Center UT Health Graduate School of Biomedical Sciences, Houston, Texas.

## Abstract

The arachidonic acid pathway participates in immunosuppression in various types of cancer. Our previous observation detailed that microsomal prostaglandin E2 synthase 1 (mPGES-1), an enzyme downstream of cyclooxygenase 2 (COX-2), limited antitumor immunity in melanoma; in addition, genetic depletion of mPGES-1 specifically enhanced immune checkpoint blockade therapy. The current study set out to distinguish the roles of mPGES-1 from those of COX-2 in tumor immunity and determine the potential of mPGES-1 inhibitors for reinforcing immunotherapy in melanoma. Genetic deletion of mPGES-1 showed different profiles of prostaglandin metabolites from that of COX-2 deletion. In our syngeneic mouse model, mPGES-1–deficient cells exhibited similar tumorigenicity to that of COX-2–deficient cells, despite a lower ability to suppress PGE2 synthesis by mPGES-1 depletion, indicating the presence of factors other than PGE2 that are likely to regulate tumor immunity. RNA-sequencing analysis revealed that mPGES-1 depletion reduced the expressions of collagen-related genes, which have been found to be associated with immunosuppressive signatures. In our mouse model, collagen was reduced in mPGES-1–deficient tumors, and phenotypic analysis of tumor-infiltrating lymphocytes indicated that mPGES-1–deficient tumors had fewer TIM3^+^ exhausted CD8^+^ T cells compared with COX-2–deficient tumors. CAY10678, an mPGES-1 inhibitor, was equivalent to celecoxib, a selective COX-2 inhibitor, in reinforcing anti-PD-1 treatment. Our study indicates that mPGES-1 inhibitors represent a promising adjuvant for immunotherapies in melanoma by reducing collagen deposition and T-cell exhaustion.

**Significance:**

Collagen is a predominant component of the extracellular matrix that may influence the tumor immune microenvironment for cancer progression. We present here that mPGES-1 has specific roles in regulating tumor immunity, associated with several collagen-related genes and propose that pharmacologic inhibition of mPGES-1 may hold therapeutic promise for improving immune checkpoint–based therapies.

## Introduction

Melanoma, which originates epidermal melanocytes, is the deadliest type of skin cancer. In fact, patients with melanoma who develop distant metastases have a dismal prognosis, with a 5-year survival rate of less than 25% (1, 2). Recent advances in drug discovery have led to new molecular targeted therapies, therapeutic vaccines, adoptive T-cell therapy, and immune checkpoint inhibitors (ICI), all of which have revolutionized treatment for melanoma (3, 4). In particular, the advent of ICIs has reaffirmed the potential role of tumor immunity, as a subset of patients has gained durable responses and long-term survival benefits from ICIs (5–7). However, a certain number of patients will experience primary resistance, and the majority of patients will develop acquired resistance regardless of their initial response to ICIs (8). It was recently shown that these resistance mechanisms are largely dependent on the negative switching of tumor-immune phenotypes induced by ICIs, such as T-cell exhaustion, the activation and stimulation of regulatory T cells, myeloid-derived suppressor cells, and the repolarization of macrophages into the M2 phenotype (9, 10). Therefore, many efforts are underway to develop effective strategies that can enhance immunotherapy by favorably altering the tumor immune microenvironment (TIME).

Various molecular components of the inflammatory process unquestionably contribute to the establishment of an immune-suppressive network in melanoma (11). Arachidonic acid metabolite pathways trigger inflammatory responses and consequently facilitate immunosuppression in the TIME (12–14). In particular, cyclooxygenase 2 (COX-2) is a well-known proinflammatory enzyme responsible for the production of multiple prostaglandins, including prostaglandin E2 (PGE2), which plays a central role in regulating the mechanisms of immune tolerance in cancer (15–20). Recently, seminal studies by Zelenay and colleagues demonstrated that genetic ablation of COX-2 reinforced the antitumor type I immune response and the accumulation of Batf2-dependent CD103^+^ dendritic cells (DC) in the TIME in a mouse model of Braf^V600E^ melanoma (15, 21). In addition, they showed that pharmacologic inhibition of COX-2 synergized with anti-PD-1 therapy and represented a promising adjuvant for immunotherapies (15). However, the specific COX-2 inhibitors are associated with an increased risk for major adverse cardiovascular events (22, 23). Therefore, our group has targeted microsomal prostaglandin E2 synthase 1 (mPGES-1; refs. 24, 25), a downstream PGE2 synthase, for which an inhibitor is likely to avoid cardiotoxicity and is expected to use in a clinical setting. Our earlier report showed that elevated mPGES-1 expression in tumor samples was linked with low CD8^+^ T-cell infiltration and unfavorable survival outcomes in patients with stage III melanoma (25). In addition, genetic depletion of mPGES-1 significantly slowed the growth of Braf^V600E^ murine melanoma tumors via the increased infiltration of CD8a^+^ T cells and CD8a^+^ DCs and thus improved anti-PD-1 efficacy in a syngeneic mouse model (25). In the current study, our goals were to distinguish the roles of mPGES-1 from those of COX-2 in tumor immunity and to directly test the potential of mPGES-1 inhibitors as an adjuvant to immune-based therapies for melanoma in a preclinical mouse model.

## Materials and Methods

### Cell Lines and Cell Culture

Human melanoma cell line WM793 (Coriell, catalog no. WC00062, RRID: CVCL_8787) was purchased from the ATCC. Murine Braf^V600E^ melanoma cells established from a C57BL/6 Braf^+/LSL-V600E^;Tyr::CreERT2^+/o^;p16^INK4a-/−^ mouse was generously provided by Dr. S. Zelenay (The University of Manchester, Manchester, England; refs. 15, 21). Cell lines were cultivated in DMEM supplemented with 10% heat-inactivated FBS, 100 U/mL penicillin, and 100 μg/mL streptomycin. Cell cultures were maintained in 5% CO_2_ at 37°C. All the cell lines had been authenticated using short tandem repeat DNA fingerprinting using the AmpFLSTR Identifiler kit, within the last 3 years. All experiments were performed with *Mycoplasma*-free cell lines.

### Knockout of Gene Expression via CRISPR/CAS9

Knockout (KO) of *ptgs1*, *ptgs2*, and *ptges* genes coding for COX-1, COX-2, and mPGES-1 proteins, respectively, was performed using CRISPR/CAS9 knockout kits (OriGene Technologies, catalog no. KN314182 for *ptgs1*, KN314183 for *ptgs2*, and KN314172 for *ptges*) according to the manufacturer's specifications. Stable KO cells were selected with puromycin, and subclones were obtained with the limiting dilution method. KO of *ptgs1*, *ptgs2*, and *ptges* genes were confirmed by measuring the mRNA and protein levels using qRT-PCR and Western blot analysis, respectively.

### ELISA

Cells were seeded in 6-well culture plates at 2 × 10^5^ cells/well. After incubating for 24 hours, cells were treated with two different concentrations of celecoxib or mPGES-1 inhibitor CAY10678 (Cayman Chemical) or 0.1% DMSO and incubated for another 24 hours. Cell culture supernatants were collected and centrifuged at 3,000 × *g* for 10 minutes. Tumor lysates (100 mg) were washed three times with PBS and prepared in 500 μL RIPA buffer supplemented with 1% protease inhibitor cocktail and 1% phosphatase inhibitor cocktail (Thermo Fisher Scientific). Then, the tissue samples were sonicated with a sonicator thoroughly on ice, followed by centrifugation at 10,000 × *g* for 20 minutes. Protein concentration was determined, and the tumor tissue lysates were analyzed for ELISA. Levels of released prostaglandins and thromboxane in supernatants and tumor tissues were quantified using Prostaglandin E2 ELISA kit, Prostaglandin D2 ELISA kit, Prostaglandin F2α ELISA kit, 6-keto Prostaglandin F1α ELISA kit, and Thromboxane B2 ELISA kit (Cayman Chemical), according to the manufacturer's recommendations.

### qRT-PCR

Total RNA was isolated with the RNeasy Mini Kit (Qiagen) according to the manufacturer's instructions. cDNA was synthesized with iScript Reverse Transcription Supermix for RT-PCR (Bio-Rad) according to the manufacturer's recommendations. Then, qRT-PCR was performed using specific oligonucleotide primers for murine *gapdh*, *ptgs1*, *ptgs2*, *ptges*, *col3a1*, *col4a1*, *col4a2*, *col8a1*, *col16a1*, and *col18a1* genes (all purchased from Qiagen). Murine *gapdh* served as a reference gene. Amplification products were detected with Applied Biosystems Fast SYBR Green Master Mix (Thermo Fisher Scientific). PCR was performed with the following protocol using Mastercycler Realplex (Eppendorf): initial denature for 60 seconds at 95°C, amplification with 40 cycles of 10 seconds at 95°C for DNA denature and 30 seconds at 60°C for primer annealing and extension. The relative fold gene expression of samples was calculated using the 2^−ΔΔ^*^C^*_t_ method.

### Western Blotting

Total proteins were extracted with RIPA buffer supplemented with 1% protease inhibitor cocktail and 1% phosphatase inhibitor cocktail. Protein aliquots were electrophoresed on either Novex WedgeWell 10% for COX-1, COX-2, and GAPDH or 16% gel for mPGES-1 (Thermo Fisher Scientific), followed by transfer to nitrocellulose membranes. After blocking with 5% nonfat milk for 1 hour at room temperature, membranes were incubated overnight at 4°C with primary Abs (1:1,000 for COX-1 Ab (clone D2G6, Cell Signaling Technology, catalog no. 9896, RRID:AB_10860249), 1:200 for COX-2 Ab (clone 33, BD Biosciences, catalog no. 610203, RRID:AB_397602), 1:250 for mPGES-1 Ab (Novus Biologicals), and 1:3,000 for GAPDH Ab (clone 6C5, Santa Cruz Biotechnology). Subsequently, membranes were incubated with IgG horseradish peroxidase (HRP)-linked secondaryAbs (1:1,000, R&D Systems) for 1 hour at room temperature. Enzymatic signals were visualized with SuperSignal West Pico Chemiluminescent Substrate (Thermo Fisher Scientific). The membranes were stripped with 2% SDS for 30 minutes at 60°C and reincubated with specific Abs as needed.

### Cell Proliferation Assay

Melanoma cells were seeded in 96-well culture plates at 4 × 10^3^ cells/well and incubated for 24 hours. Then, cells were exposed to different concentrations of celecoxib and CAY10678. After 24, 48, and 72 hours of treatment, cells were incubated with PrestoBlue Cell Viability Reagent (Thermo Fisher Scientific) for 2 hours according to the manufacturer's recommendations. The absorbance was monitored with a spectrophotometer at 570 nm using 600 nm as a reference wavelength.

### *In Vivo* Experiments

The animal experimental protocol was approved by the Institutional Animal Care and Use Committee at MD Anderson. All animal experiments were performed according to the NIH guidelines. Six-week-old female C57BL/6J mice, purchased from the Jackson Laboratory, were anesthetized with isoflurane and were subcutaneously inoculated 100 μL suspensions of murine Braf^V600E^ melanoma cells (wild type, scramble control, *ptgs1* KO, *ptgs2* KO, and *ptges* KO) into the right flank (1 × 10^6^ per mouse). In treatment-control experiments, 8 days after cell injection, mice were randomly divided into two groups for the first study: vehicle or CAT10678, and six groups for the second study: IgG control, anti-PD-1 Ab (αPD-1), celecoxib+IgG, CAY10678+IgG, celecoxib+αPD-1, or CAY10678+αPD-1. αPD-1 (200 μg per mouse, BE0416, Bio X Cell) or IgG control (BE0086, Bio X Cell) was intraperitoneally administered every 3 days for a total of four times, and 50 mg/kg celecoxib or 100 mg/kg CAY10678, suspended with 1% Tween 80 and 0.5% carboxymethylcellulose in normal saline, was intraperitoneally administered daily for 13 days. Tumor length (*L*) and width (*W*) were measured every 3 or 4 days with an external caliper, and the volume (*V*) of each tumor was calculated according to the equation [*V* = (*L* × *W*^2^) × 0.5]. Body weight was also measured at the same time. The mice were euthanized by carbon dioxide asphyxiation. Tumors were then excised, weighed, and subjected to Masson's trichrome staining or multiplex fluorescent IHC (mfIHC) staining.

### Imaging Analysis and Selection of Regions of Interest

All sections were scanned using Vectra Polaris Imaging System (Akoya Biosciences). At least two and up to five of regions of interest (ROI; each ROI was 1.1×1.5 mm) were randomly selected from each mouse tumor, depending on the tumor size, using InForm software (PerkinElmer) for the quantitative analysis of collagen deposition and tumor-infiltrating lymphocyte (TIL) distribution (Supplementary Fig. S1A). ROIs for the analysis of TIL distribution were selected at the same location as those for the collagen deposition analysis (Supplementary Fig. S1B).

### Masson's Trichrome Staining and Quantitative Analysis of Collagen Deposition

To evaluate the collagen deposition in mouse tumor tissues, sections of formalin-fixed paraffin-embedded (FFPE) tissues with a thickness of 5 μm were stained by Masson's trichrome stain kit (Polysciences Inc.) according to the manufacturer's recommendations. Collagen positivity (%) was calculated using automated digital pathology and image analysis software (Visiopharm). On each ROI, background glass and vessel area were excluded from analysis and collagen positivity (%) was automatically calculated as the ratio of the Masson's trichrome–positive area to the entire area (Supplementary Fig. S2).

### mfIHC Staining

mfIHC staining was performed using Opal 7-Color Manual IHC Kit (Akoya Biosciences), according to the manufacturer's recommendations. Concretely, 5 μm FFPE tissues were baked for 60 minutes at 60°C. After deparaffinization, a heat-mediated stripping procedure using AR6 buffer (Akoya Biosciences) at 95°C for 15 minutes was performed with the EZ-Retriever systems V.3 (BioGenex) between each Ab staining cycle. After blocking with Protein Block (Agilent Technologies Inc) for 20 minutes, slides were incubated for 30 minutes at room temperature with primary Abs. Sequentially, Opal Polymer HRP Ms + Rb was introduced for 10 minutes and preselected opal fluorophores were applied for 10 minutes at room temperature. The staining procedure was carried out in a step-by-step manner. After all staining was completed, slides were counterstained with 4′,6-diamidino-2-phenylindole (DAPI) solution (Supplementary Fig. S3A–S3D). Supplementary Table S1 contains the Abs and reagents for the four panels utilized.

### Quantitative Analysis of Tumor-infiltrating Immune Cells

The number of signal-positive c CD45^+^CD8a^+^IFNγ^+^ells was counted using Visiopharm software. First, each cell was stained with DAPI and segmented using the software's nuclei detection application, and the total number of cells in each ROI was automatically counted. Next, the signal positivity threshold for each marker was manually optimized on a one-by-one basis, and the number of signal-positive or -negative cells was automatically counted in a step-by-step manner (Supplementary Fig. S4). By this method, a total of 10 cell lineages were assessed from four mfIHC panels (Supplementary Table S2).

### RNA Sample Processing and Sequencing

The extracted RNA from *ptgs1-*KO, *ptgs2-*KO, *ptges-*KO, and scramble cells using the RNeasy Mini Kit were checked for overall quality. Only RNA samples with high quality (RNA Integrity Number [RIN] > 8.0) and high purity (Optical Density [OD] 260/280 = 1.8–2.0) were used. mRNA libraries were generated with a Bioo Scientific NEXTflex Rapid Directional RNA sequencing (RNA-seq) Library Prep Kit. The mRNA libraries were then sequenced on an Illumina HiSeq 2500 in rapid mode using 101 bp paired-end reads.

### Bioinformatic Analysis

RNA-seq normalized data were utilized to compare *ptgs1*, *ptgs2*, or *ptges* mRNA levels across all the control (scramble) and KO conditions. Of the 28 types of collagens encoded by 43 genes in the genome, only collagen genes with average normalized counts greater than 10 (*col3a1, col4a1, col4a2, col4a5, col5a1, col8a1, col16a1,* and *col18a1*) were included and analyzed by principal component analysis (PCA) and heat maps using ClustVis software (ClustVis, RRID:SCR_017133).

RNA-seq expression data for The Cancer Genome Atlas (TCGA)-Skin Cutaneous Melanoma (SKCM) were obtained from https://xenabrowser.net/ in May 2022. All the data were normalized to log_2_ normalized counts+1. Only patients with metastatic melanoma from TCGA-SKCM were included in the analysis. Patients with metastatic melanoma were divided into four quantiles based on *PTGES* mRNA levels. Patients in the lower quartile (Low-*PTGES*, *n* = 92) and the upper quartile (High-*PTGES*, *n* = 92) were included and compared for *PTGES, COL1A1, COL1A2, COL3A1, COL4A1, COL4A2, COL8A1, COL16A1,* and *COL18A1* mRNA levels.

### Statistical Analysis

Statistical analyses were conducted using GraphPad Prism 8 (GraphPad Software Inc., RRID:SCR_002798) or R version 4.1.2 (https://www.R-project.org/) in a two-tailed way. Continuous variables were expressed as mean ± 95% confidence interval. Statistical differences between two groups were compared using Student *t* test. Multiple groups were analyzed by one-way ANOVA and Tukey multiple comparisons test. Chronologic changes in tumor volume between two groups were compared by repeated measures one-way ANOVA, followed by Tukey *post hoc* test. Survival curves were estimated via the Kaplan–Meier method and compared via the log-rank test. *P* values < 0.05 were considered statistically significant.

### Data Availability

The RNA-seq data generated and discussed in this study have been deposited in the NCBI Gene Expression Omnibus (GEO) and are accessible through the GEO Series accession number GSE236204.

## Results

### COX-1, COX-2, or mPGES-1 Expression Associates with Different Prostaglandin Profiles in Murine Melanoma Cells

**FIGURE 1:**
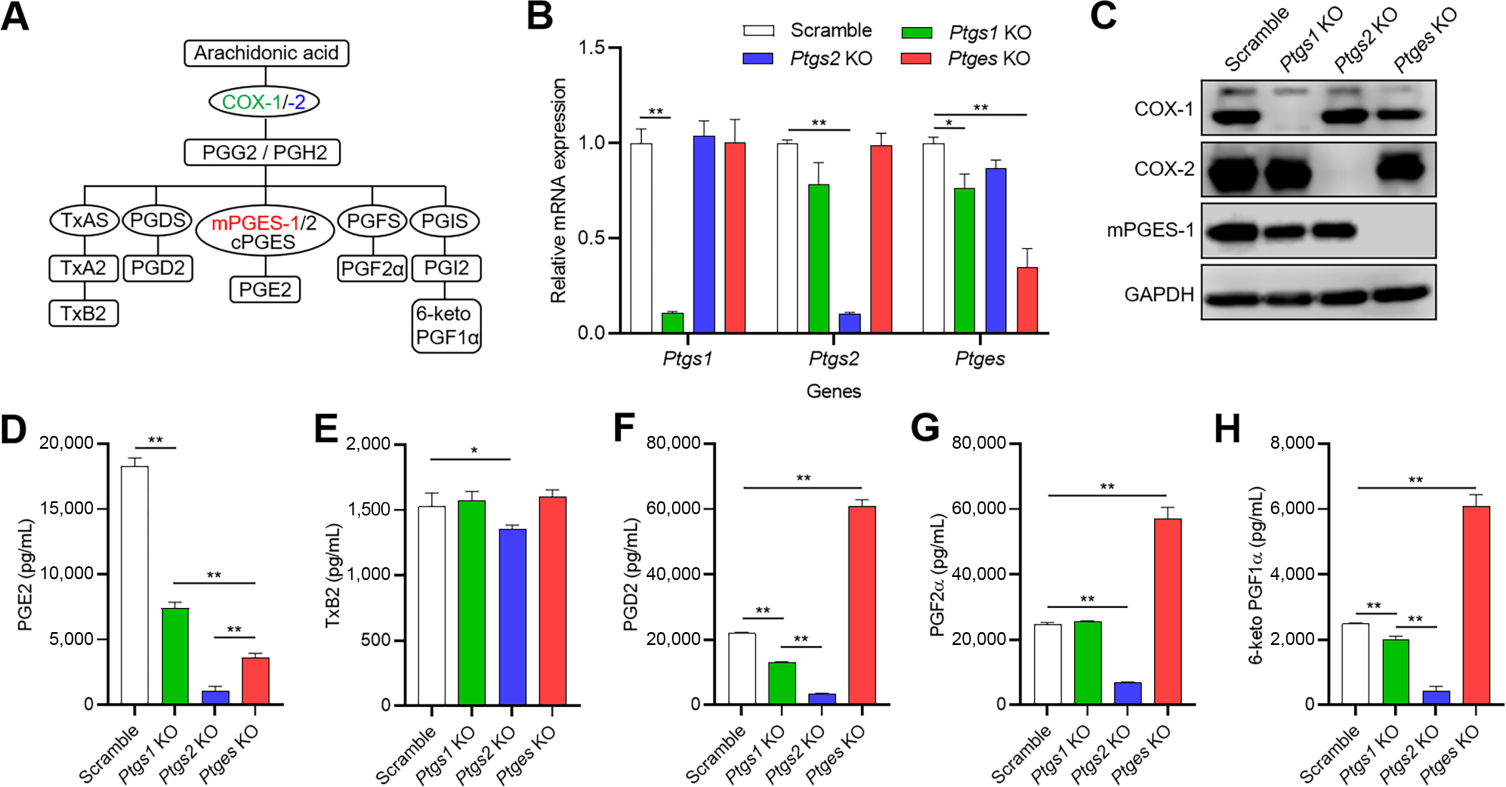
The influence of *ptgs1*, *ptgs2*, and *ptges* KO on the production of arachidonic acid metabolites from murine Braf^V600E^ melanoma cells. **A,** Schematic diagram of the arachidonic acid metabolic cascade. PGG2, prostaglandin G2; PGH2, prostaglandin H2; TxAS, thromboxane-A synthase; TxA2, thromboxane A2; PGDS, prostaglandin D synthase; cPGES, cytosolic prostaglandin E synthase; PGFS, prostaglandin F synthase; PGIS, prostaglandin I synthase. **B** and **C,***ptgs1*, *ptgs2*, and *ptges* genes in murine Braf^V600E^ melanoma cells were knocked out via CRISPR/CAS9. The mRNA and protein levels were analyzed by qRT-PCR (B) and Western blotting (C). Bar graphs show the fold change relative to mRNA levels of scramble control for each gene (B, *n* = 4). GAPDH was used as a loading control (C). **D–H,** Bar plot showing the concentrations of arachidonic acid metabolites released in supernatants obtained from scramble control, *ptgs1-*KO, *ptgs2-*KO, and *ptges-*KO cells. All prostanoids were measured by ELISA (*n* = 4): PGE2 (D), TxB2 (E), PGD2 (F), PGF2α (G), and 6-keto PGF1α (H). Graph values represent mean ± SD. Significance in difference between two groups was determined by Student *t* test. **, *P* < 0.01; *, *P* < 0.05.

Several enzymes convert the arachidonic acid present in the phospholipids of cell membranes into active metabolites (Fig. 1A). To evaluate the specific roles of mPGES-1 in melanoma, we established murine Braf^V600E^ melanoma cells, which knocked out *ptgs1*, *ptgs2*, and *ptges* genes (coding for COX-1, COX-2, and mPGES-1 proteins, respectively), using CRISPR/CAS9. As reported previously, murine Braf^V600E^ melanoma cells have elevated levels of COX-2 and mPGES-1 and produce a large amount of prostaglandins in normal conditions (15, 24). KO efficacy for *ptgs1*, *ptgs2*, and *ptges* genes and corresponding proteins were respectively assessed by qRT-PCR and Western blotting (Fig. 1B and C). Then, we compared the prostaglandin and thromboxane levels released in supernatants. As thromboxane A2 (TxA2) and prostaglandin I2 (PGI2) are highly labile and rapidly degraded, we instead measured thromboxane B2 (TxB2) and 6-keto prostaglandin F1α (PGF1α), respectively (Fig. 1A). Suppression of PGE2 release was most effective with *ptgs2* KO and moderately effective with *ptges* and *ptgs1* KO (Fig. 1D). Deletion of *ptgs2* gene slightly but significantly decreased the release of TxB2 (Fig. 1E). The release of prostaglandin D2 (PGD2), prostaglandin F2α (PGF2α), and 6-keto PGF1α were dramatically reduced by *ptgs2* KO and slightly decreased or unchanged by *ptgs1* KO. In contrast, *ptges* KO dramatically increased PGD2, PGF2α, and 6-keto PGF1α formation (Fig. 1F–H).

### mPGES-1 and COX-2 Deletion Comparably Suppresses Tumor Growth in a Syngeneic Mouse Model

**FIGURE 2:**
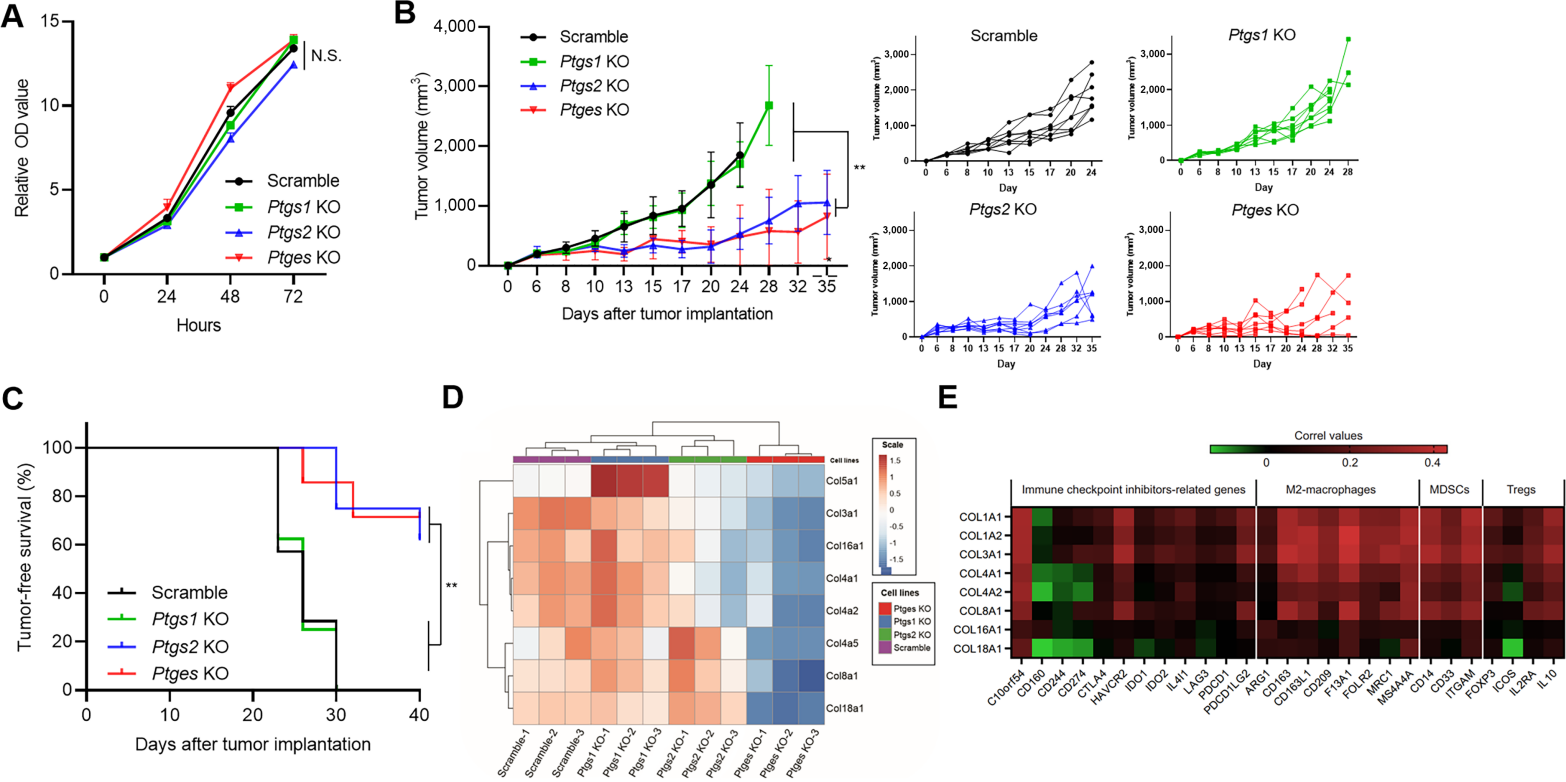
*In vivo* tumor growth assessment and RNA-seq characterization of murine Braf^V600E^ melanoma cells with *ptgs1*, *ptgs2*, or *ptges* KO. **A,** Cell proliferation assay in scramble, *ptgs1-*KO, *ptgs2*-KO, and *ptges*-KO murine Braf^V600E^ melanoma cells. Results represent the fold change relative to the OD value of baseline levels at time 0 for each cell (0, 24, 48, and 72 hours after culture; *n* = 6). Differences between two groups were analyzed by repeated measures one-way ANOVA, followed by Tukey *post hoc* test. **B,** Left, Tumor volume measurement at indicated timepoints for scramble, *ptgs1*-KO, *ptgs2-*KO, and *ptges*-KO cells. A total of 1 × 10^6^ cells per mouse were injected subcutaneously into the flanks of C57BL/6J mice: *n* = 8 mice each for scramble, *ptgs1-*KO, and *ptgs2*-KO groups, and *n* = 7 for *ptges*-KO group. Differences in tumor volume between two groups were analyzed by repeated measures one-way ANOVA, followed by Tukey *post hoc* test. **B,** Right, Tumor volumes for individual mice in each group are shown. **C,** Tumor-free survival (tumor volume < 1,000 mm^3^) was estimated using the Kaplan–Meier method and compared using the log-rank test. **D,** Heat map showing the unsupervised hierarchical clustering of the mRNA levels (Reads Per Kilobase Million [RPKM]) of eight collagen-related genes across scramble, *ptgs1*-KO, *ptgs2-*KO, and *ptges-*KO cells. RNA was extracted from three different subclones in each cell line. **E,** Spearman correlation values between immune-suppressive markers and collagen-related genes in melanoma samples from TCGA-SKCM dataset. Graph values represent mean ± SD. **, *P* < 0.01. N.S., not statistically significant.

To elucidate the impact of COX-1, COX-2, and mPGES-1 expression on tumor burden, we implanted *ptgs1-*KO, *ptgs2-*KO, *ptges-*KO, or scramble cells into the flank of syngeneic immunocompetent C57BL/6J mice and compared the tumor growth. Before cell implantation, four subclones of *ptgs1-*KO, *ptgs2-*KO, *ptges-*KO, and scramble cells with similar proliferation rates *in vitro* were selected (Fig. 2A). The tumor growth originating from *ptgs2-* or *ptges-*KO cells was significantly slower than that of tumors originating from scramble or *ptgs1*-KO cells (Fig. 2B), whereas the tumor growth was not significantly different between *ptgs1* KO*-* and scramble-derived tumors and between *ptgs2* KO*-* and *ptges* KO*-*derived tumors. Moreover, tumor-free survival (tumor volume < 1,000 mm^3^) was longer in *ptgs2* KO*-* and *ptges* KO*-*derived tumors compared with *ptgs1* KO*-* and scramble cell-derived tumors (Fig. 2C).

### mPGES-1 Depletion Suppresses Expression of Collagen-related Genes in Murine Melanoma Cells

The similar tumorigenicity between *ptgs2-* and *ptges-*KO cells (Fig. 2B) despite their difference in PGE2 suppression potencies (Fig. 1D) suggests the presence of other factors which may specifically be involved in the regulation of tumor progression and immune cell responses by mPGES-1. To address this, we performed RNA-seq analysis using three independent samples from each scramble, *ptgs1-*KO, *ptgs2-*KO, and *ptges*-KO murine Braf^V600E^ melanoma cells cultured *in vitro*. The mRNA levels of *ptgs1* showed a decreased in *ptgs1*-KO, but not *ptgs2-*KO or *ptges-*KO cells compared with scramble control cells (Supplementary Fig. S5A). Similarly, mRNA levels of *ptgs2* were reduced in *ptgs2-*KO cells, but not in *ptgs1-*KO or *ptges-*KO cells, compared with scramble cells (Supplementary Fig. S5B). Also, the mRNA levels of *ptges* were reduced in *ptgs1*-KO and *ptges-*KO cells, but not *ptgs2-*KO cells, compared with scramble cells (Supplementary Fig. S5C). In summary, all these results validated the results observed in qRT-PCR and Western blot analysis (Fig. 1B and C). We focus on analyzing genes that code for collagen molecules, a total of 43 genes in the human/mouse genome. In the RNA-seq dataset, only collagen genes with average normalized counts greater than 10 (*col3a1*, *col4a1*, *col4a2*, *col4a5*, *col5a1*, *col8a1*, *col16a1*, and *col18a1*) were included and analyzed. In PCA, we observed that the eight collagen genes clustered each of the cell line conditions analyzed (Supplementary Fig. S5D). Also, heat map (Fig. 2D) and boxplots (Supplementary Fig. S6A–S6H) showed that all the eight collagen genes were significantly downregulated in *ptges-*KO cells compared with scramble control cells. RNA-seq data were validated by qRT-PCR. Although some qRT-PCR results were inconsistent with RNA-seq data, mRNA levels of some of these genes, including *col3a1*, *col4a1*, *col4a2*, *col8a1*, *col16a1*, and *col18a1* were reduced in *ptges-*KO cells compared with scramble cells (Supplementary Fig. S6I and S6J). To evaluate the clinical relevance of this finding, we assessed a cohort of patients with metastatic melanoma from TCGA-SKCM dataset. Patients with high and low mRNA levels of *PTGES* (*n* = 92 each) were compared (Supplementary Fig. S7A). In accordance with the results of mouse cells, mRNA levels of collagen-related genes *COL3A1*, *COL4A1*, *COL4A2*, *COL8A1*, and *COL18A1* were significantly lower in patients with low *PTGES* than in those with high *PTGES* (Supplementary Fig. S7B–S7F). In contrast, *COL16A1* levels were similar between the two groups (Supplementary Fig. S7G). Intriguingly, mRNA levels of *COL1A1* and *COL1A2*, coding for collagen type 1, which is most abundant of the collagens, were also linked with *PTGES* in patients with melanoma (Supplementary Fig. S7H and S7I). Data integration from RNA-seq, qRT-PCR, and TCGA-SCKM RNA-seq showed that four collagen genes (*col3a1, col4a1, col4a2,* and *col8a1*) were consistently downregulated when the *ptges* mRNA levels were decreased (Supplementary Fig. S6J). Furthermore, *ptges-*KO murine Braf^V600E^ melanoma cells, cultivated *in vitro*, were less aggregated than scramble, *ptgs1*-KO, and *ptgs2-*KO cells (Supplementary Fig. S8A). Although different cell aggregation may be influenced by different expression of cell-cell adhesion molecules, *ptges-*KO cells cultured in collagen IV-coated well (Thomas Scientific) were more aggregated than those cultured in normal well (Supplementary Fig. S8B), suggesting that *ptges*-KO cells released the lowest amount of collagen which was partially associated with cell aggregation. Of note, cutaneous melanoma data obtained from the TCGA-SKCM dataset demonstrated that mRNA levels of some collagen-related genes were closely correlated with immunosuppressive signatures (Fig. 2E). These results suggest that mPGES-1 regulates the production and release of collagen, which may be associated with immune suppression in melanoma.

### Differences in Collagen Deposition and TIL Distribution Between Tumors Derived from COX-2– and mPGES-1–depleted Cells

**FIGURE 3:**
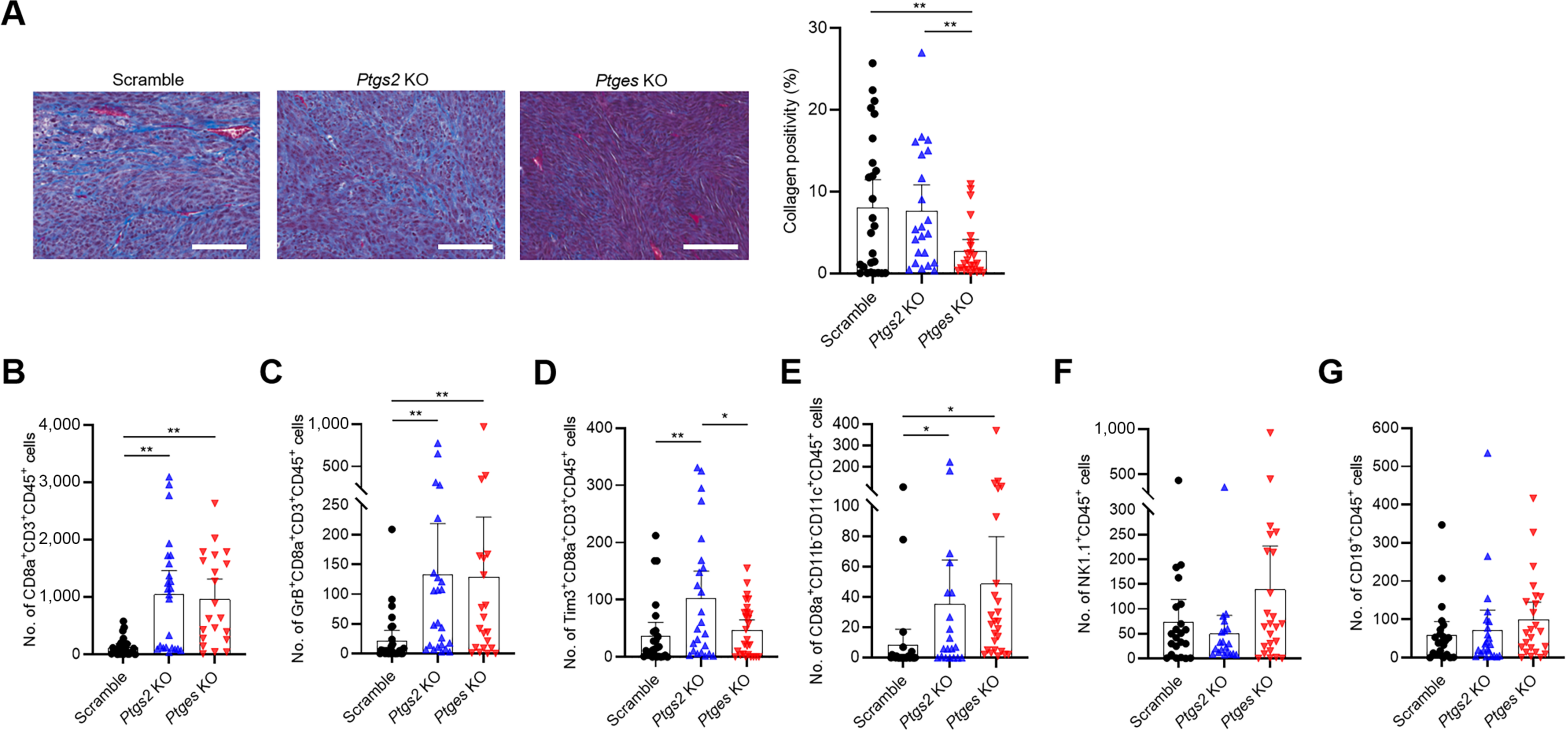
Collagen deposition and distribution of tumor-infiltrating immune cells in tumors derived from *ptgs2*-KO and *ptges*-KO murine Braf^V600E^ melanoma cells. **A,** Left, Representative images of Masson's trichrome staining of tumors derived from scramble, *ptgs2*-KO, and *ptges*-KO cells. Scale bar = 100 μm. **A,** right, Collagen positivity (%) was automatically calculated using Visiopharm software. **B–G,** Distribution of tumor-infiltrating immune cells was compared between tumors from scramble, *ptgs2*-KO, and *ptges-*KO cells. The number of tumor-infiltrating immune cells was automatically calculated using Visiopharm software. Shown are the numbers of tumor-infiltrating CD8a^+^ cytotoxic T cells (B), tumor-infiltrating GrB^+^ effector CD8a^+^ T cells (C), tumor-infiltrating Tim3^+^ exhausted CD8a^+^ T cells (D), tumor-infiltrating classic CD8a^+^ DCs (E), tumor-infiltrating NK1.1^+^ NK cells (F), and tumor-infiltrating CD19^+^ B cells (G). Two to five ROIs, depending on tumor size, were randomly selected from each tumor and used for analyses. Graph values represent mean ± SD. Significance in difference between two groups was determined by Student *t* test. **, *P* < 0.01; *, *P* < 0.05.

To identify the distinctive role of intrinsic mPGES-1 from that of COX-2 at the tumor site, we compared the collagen deposition and TIL distribution among tissues from similarly sized tumors derived from scramble, *ptgs2-*KO, and *ptges*-KO murine Braf^V600E^ melanoma cells. First, we performed Masson's trichrome staining to compare the intratumoral collagen deposition across the three groups. Collagen positivity was significantly diminished by *ptges* KO, but it was not altered by *ptgs2* KO (Fig. 3A). Next, the distribution of TILs was assessed by mfIHC staining (Supplementary Fig. S9A–S9F and Supplementary Fig. S10A–S10C). Tumor-infiltrating cytotoxic CD8a^+^ T cells were drastically increased by *ptgs2* and *ptges* KO; however, no significant difference was observed between tumors derived from *ptgs2-*KO and *ptges-*KO cells (Fig. 3B; Supplementary Fig. S9A).

Subsequently, we analyzed effector and exhaustion markers of cytotoxic CD8a^+^ T cells. The total number of tumor-infiltrating granzyme B^+^ (GrB^+^) effector CD8a^+^ T cells was greatly improved by *ptgs2* and *ptges* KO, although the percentage of GrB^+^CD8a^+^ T cells in total CD8a^+^ T cells was similar between three groups and no significant difference was observed between *ptgs2-*KO and *ptges-*KO cells (Fig. 3C; Supplementary Fig. S9B). The number of infiltrating IFNγ^+^CD8a^+^ T cells was significantly higher in tumors derived from *ptges*-KO versus scramble cells (Supplementary Fig. S10A). Notably, the number and frequencies of tumor-infiltrating Tim3^+^ exhausted CD8a^+^ T cells were significantly fewer in tumors derived from *ptges*-KO versus scramble and *ptgs2-*KO cells (Fig. 3D; Supplementary Fig. S9C). Conversely, the number of infiltrating IFNγ^+^Tim3^−^CD8a^+^ T-cell population was significantly higher in tumors derived from *ptges*-KO cells compared with scramble and *ptgs2*-KO cells (Supplementary Fig. S10B). The number of Tim3^+^PD-1^+^LAG3^+^ exhausted CD8a^+^ T cells was lowest in *ptges*-depleted cell-derived tumors, followed by *ptgs2*-depleted and scramble cell-derived tumors although the difference did not reach statistically significant (Supplementary Fig. S10C). Furthermore, we compared tumor-infiltrating classic CD8a^+^ DCs population, synonymous with Batf3^+^ DCs, because this subset is a key orchestrator of immune responses in cancer and our previous work demonstrated that *ptges* depletion could enhance this population in melanoma (25). In agreement with our earlier work, tumor-infiltrating CD8a^+^ DCs were more abundant in *ptges*-depleted cell-derived tumors compared with scramble cell-derived tumors, but this was not specific to *ptges* as *ptgs2* depletion equivalently upregulated the frequencies (Fig. 3E; Supplementary Fig. S9D). Finally, the frequencies of tumor-infiltrating natural killer (NK) and B cells were examined because tumor-derived PGE2 inhibited the accumulation of these two immune cell types in previous studies (21, 26, 27). However, in the current study, neither *ptgs2* nor *ptges* depletion affected the trafficking of NK cells and B cells into tumors (Fig. 3F and G; Supplementary Fig. S9E and S9F).

### mPGES-1 Inhibitor CAY10678 Suppresses Tumor Growth in a Syngeneic Mouse Model

**FIGURE 4:**
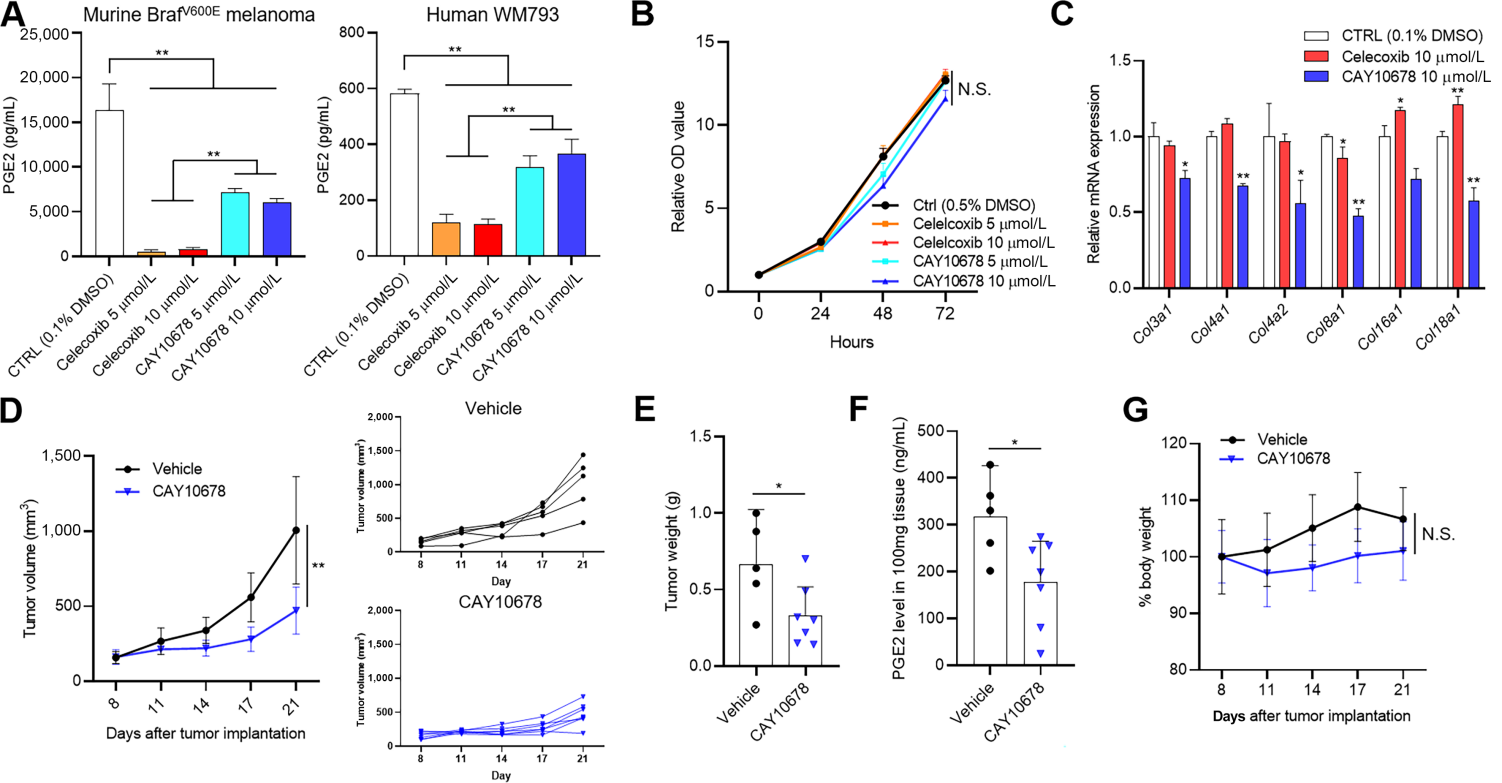
Functional differences between celecoxib and CAY10678 and the impact of CAY10678 on tumor growth in mice. **A,** Release of PGE2 in supernatants in murine Braf^V600E^ melanoma cells (left) and human WM793 cells (right) treated with different concentrations (0, 5, and 10 μmol/L) of celecoxib and CAY10678, measured by ELISA (*n* = 4). **B,** Proliferation assay of murine Braf^V600E^ melanoma cells. Cells were treated with different concentrations of celecoxib and CAY10678 (0, 5, and 10 μmol/L). Cell viability was assessed at 0, 24, 48, and 72 hours. Results represent the fold change relative to the OD value of each group prior to treatment (*n* = 6). Differences between two groups were analyzed by repeated measures one-way ANOVA, followed by Tukey *post hoc* test. **C,** Shown are results of qRT-PCR analyses for *col3a1*, *col4a1*, *col4a2*, *col8a1*, *col16a1*, and *col18a1* genes in murine Braf^V600E^ melanoma cells treated with 0.1% DMSO, 10 μmol/L celecoxib, or 10 μmol/L CAY10678 for 24 hours. Bar graphs show the fold change relative to mRNA levels of control (0.1% DMSO) for each gene (*n* = 4). **D,** Left, Tumor volume at indicated timepoints for murine Braf^V600E^ melanoma cells. A total of 1 × 10^6^ cells per mouse were injected subcutaneously into the flanks of C57BL/6J mice. The tumor-bearing mice were randomly assigned into two groups on day 8: vehicle (*n* = 5) and CAY10678 (*n* = 7) groups. Mice in the CAY10678 group were treated with intraperitoneal injection of 100 mg/kg CAY10678 daily. **D**, Right, Tumor volume for individual mice in each treatment group. **E,** Mice were euthanized on day 21, and the tumors were weighed. **F,** PGE2 levels in tumors were measured by ELISA. **G,** Chronologic changes in body weight in the two groups. Graph values represent mean ± SD. Significance in difference between two groups was tested by Student *t* test. Differences in tumor volume and body weight were analyzed by repeated measures one-way ANOVA followed by Tukey *post hoc* test. **, *P* < 0.01; *, *P* < 0.05. N.S., not statistically significant.

Slight differences in the amino acid compositions of human and mouse mPGES-1 demands the use of a mouse/human dual mPGES-1 inhibitor for preclinical *in vivo* study. CAY10678 was selected as a potential candidate to selectively inhibit recombinant human (IC_50_ = 90 nmol/L) and rat mPGES-1 (IC_50_ = 900 nmol/L; ref. 28). CAY10678 attenuated the release of PGE2 from both human WM793 and murine Braf^V600E^ melanoma cells by up to 50%, albeit to a lesser degree than celecoxib (Fig. 4A). The effect of these inhibitors on the release of other prostaglandins and thromboxane in supernatants was similar to that of genetic depletion of *ptgs2* and *ptges* in murine Braf^V600E^ melanoma cells (Fig. 1E–H); only celecoxib decreased TxB2 production (Supplementary Fig. S11A), whereas PGD2, PGF2α, and 6-keto PGF1α production were suppressed by celecoxib and increased by CAY10678 (Supplementary Fig. S11B–S11D).

Exposure to different concentrations (5 and 10 μmol/L) of celecoxib and CAY10678 did not influence proliferation of murine Braf^V600E^ melanoma cells *in vitro* (Fig. 4B). Only CAY10678 effectively reduced the mRNA levels of multiple collagen genes (Fig. 4C). In C57BL/6J mice injected with murine Braf^V600E^ melanoma cells, CAY10678 treatment significantly delayed tumor growth compared with vehicle treatment (Fig. 4D). CAY10678-treated mice had significantly lower tumor weight than untreated mice at the time of euthanasia (Fig. 4E). In addition, PGE2 levels were lower in CAY10678-treated tumor tissue versus untreated tumor tissue (Fig. 4F). Mice treated with CAY10678 displayed transient body weight loss, most relevant at 3 days of treatment, yet this change was not significant compared with untreated mice through the observation period (Fig. 4G).

### CAY10678 and Celecoxib Comparably Enhance Anti-PD-1 Therapy in a Syngeneic Mouse Model

**FIGURE 5:**
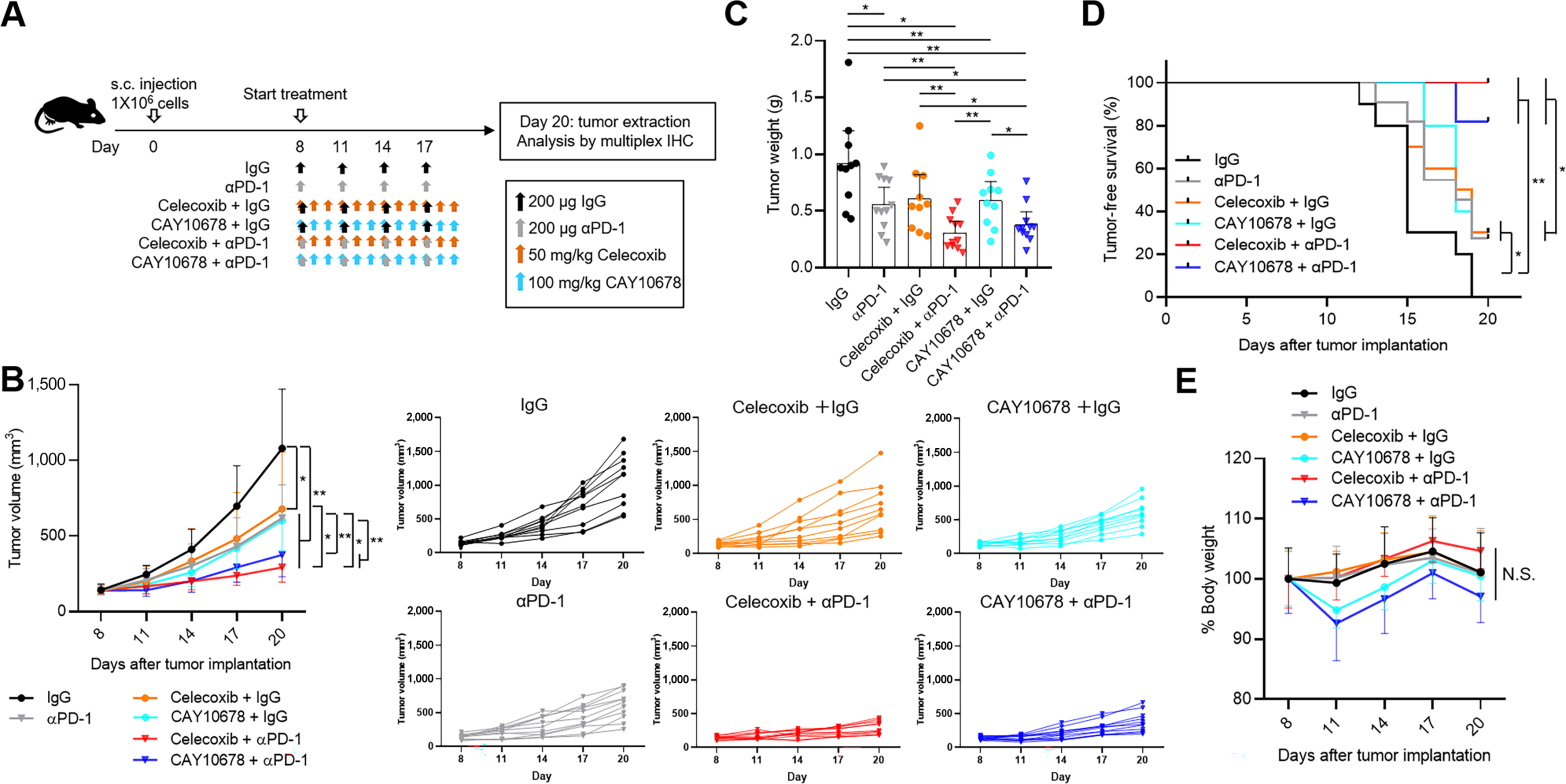
Celecoxib or CAY10678 as monotherapy and combined with PD-1 blockade. **A,** Experimental schema illustrating the six different treatment groups. A total of 1 × 10^6^ murine Braf^V600E^ melanoma cells per mouse were injected subcutaneously into the flanks of C57BL/6J mice. On day 8, tumor-bearing mice were randomly assigned into six groups; IgG (*n* = 11), αPD-1 (*n* = 12), celecoxib+IgG (*n* = 11), CAY10678+IgG (*n* = 11), celecoxib+αPD-1 (*n* = 12), and CAY10678+αPD-1 (*n* = 12). IgG or αPD-1 (200 μg per mouse) was intraperitoneally administered every 3 days, and 50 mg/kg celecoxib or 100 mg/kg CAY10678 was intraperitoneally administered daily. **B,** Left, Tumor volume at indicated timepoints for murine Braf^V600E^ melanoma cells treated with IgG, αPD-1, celecoxib+IgG, CAY10678+IgG, celecoxib+αPD-1, and CAY10678+αPD-1. **B**, Right, Tumor volume for individual mice in each treatment group. **C,** Mice were euthanized on day 20, and tumor weight was measured. **D,** Tumor-free survival (tumor volume < 500 mm^3^) was estimated using the Kaplan–Meier method and compared using the log-rank test. **E,** Chronologic changes in body weight in the six groups. Graph values represent mean ± SD. Differences in tumor volume and body weight were analyzed by repeated measures one-way ANOVA followed by Tukey *post hoc* test. Significance in difference between two groups was determined by Student *t* test **, *P* < 0.01; *, *P* < 0.05.

Our recent study demonstrated that genetic deletion of mPGES-1 enhanced PD-1 blockade in a syngeneic mouse model of Braf^V600E^ melanoma (25), indicating the potential of mPGES-1 inhibitors for improving immunotherapy. In addition, COX-2 inhibitors significantly synergized with immunotherapy in the same mouse model (15). We therefore set up a head-to-head study to directly compare the impact of mPGES-1 and COX-2 inhibitors on anti-PD-1 therapy. Eight days after inoculation with parent murine Braf^V600E^ melanoma cells, mice were randomized into six groups: IgG control, αPD-1, celecoxib+IgG, CAY10678+IgG, celecoxib+αPD-1, CAY10678+αPD-1 (Fig. 5A). Single-drug therapy with αPD-1, celecoxib, and CAY10678 moderately and similarly slowed tumor growth; suppression of tumor growth was more powerful using combinations of celecoxib or CAY10678 with αPD-1 (Fig. 5B). Tumor weight of mice treated with combination therapies was significantly lower than that of mice treated with single-drug therapies at the time of euthanasia (Fig. 5C). Tumor-free survival (tumor volume < 500 mm^3^) was most extended in mice treated with combination therapies and somewhat extended with single-drug therapies (Fig. 5D). Tumor growth speed and weight as well as tumor-free survival were all similar between mice treated with celecoxib+αPD-1 and CAY10678+αPD-1 (Fig. 5B–D). Consistent with the results of CAY10678 single-drug therapy (Fig. 4G), mice treated with CAY10678 and CAY10678+αPD-1 experienced slight body weight loss of less than 10% (Fig. 5E)

### Differences in Collagen Deposition and TIL Distribution Between Tumors from Mice Treated with Celecoxib or CAY10678 with αPD-1

**FIGURE 6:**
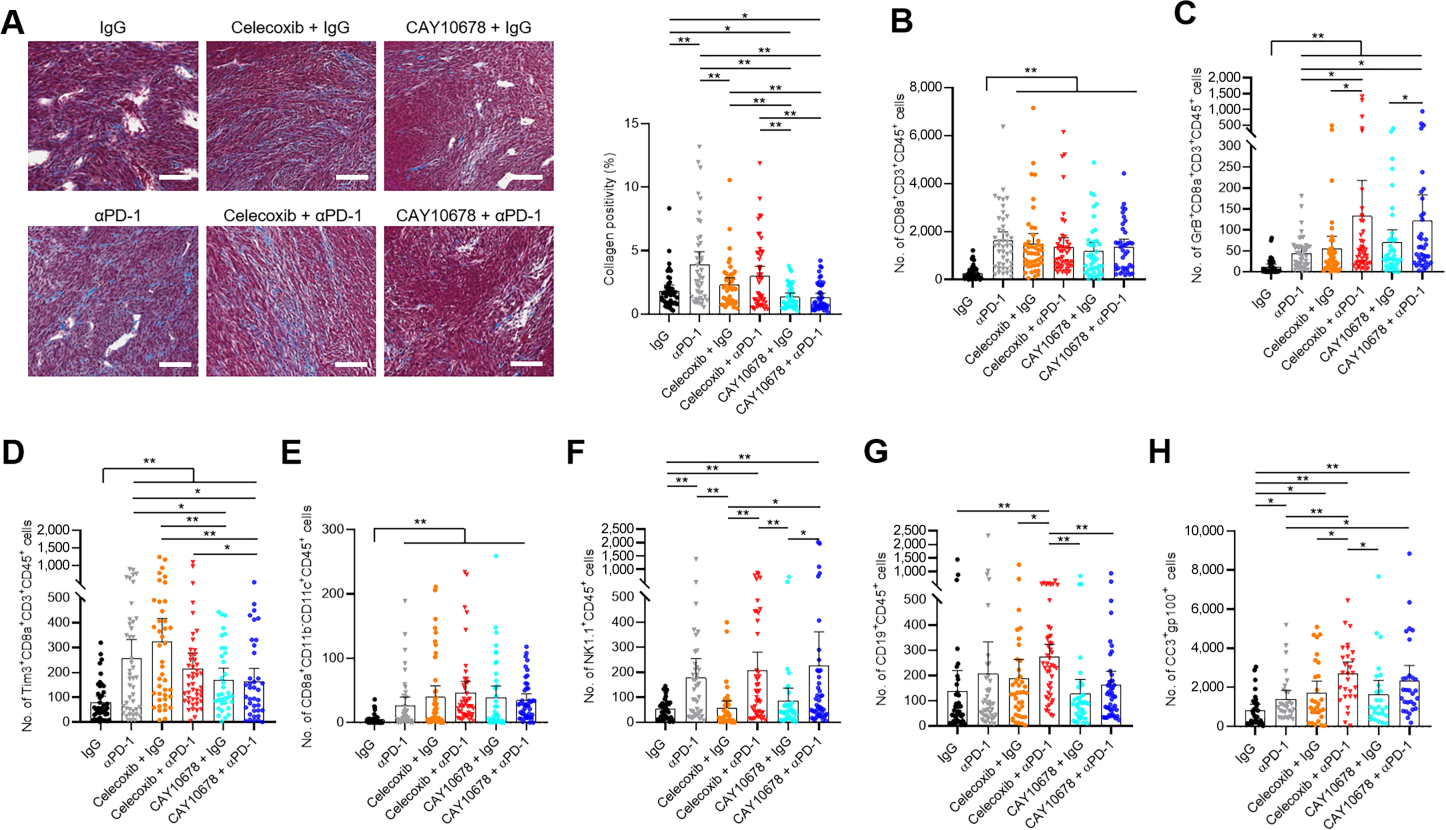
Collagen deposition and distribution of tumor-infiltrating immune cells in tumors treated with celecoxib or CAY10678 as monotherapy or combined with and PD-1 blockade. **A**, Left, Representative images of Masson's trichrome staining of tumors treated with IgG, αPD-1, celecoxib+IgG, CAY10678+IgG, celecoxib+αPD-1, and CAY10678+αPD-1. Scale bar = 100 μm. **A**, Right, Collagen positivity (%) was automatically calculated using Visiopharm software. **B–G,** Distribution of tumor-infiltrating immune cells was compared between tumors treated with IgG, αPD-1, celecoxib+IgG, CAY10678+IgG, celecoxib+αPD-1, and CAY10678+αPD-1. The number of tumor-infiltrating immune cells was automatically calculated using Visiopharm software. Shown are the numbers of tumor-infiltrating CD8a^+^ cytotoxic T cells (B), tumor-infiltrating GrB^+^ effector CD8a^+^ T cells (C), tumor-infiltrating Tim3^+^ exhausted CD8a^+^ T cells (D), tumor-infiltrating classic CD8a^+^ DCs (E), tumor-infiltrating NK1.1^+^ NK cells (F), and tumor-infiltrating CD19^+^ B cells (G). **H,** Number of cleaved caspase-3 (CC3)^+^ cells in gp100^+^ tumor cells. Two to five ROIs, depending on tumor size, were randomly selected from each tumor and used for further analyses. Graph values represent mean ± SD. Significance in difference between two groups was determined by Student *t* test. **, *P* < 0.01; *, *P* < 0.05.

Finally, we compared the collagen deposition and TIL distribution using tumors of drug-treated mice at the time of euthanasia. αPD-1 monotherapy significantly induced the collagen in tumors (Fig. 6A), which may be associated with earlier findings that long-term exposure to αPD-L1 accelerated intratumoral collagen deposition in a murine lung cancer model (29). In contrast, CAY10678 effectively reduced collagen deposition, even when combined with αPD-1. Consequently, the difference of TIL distribution was evaluated with mfIHC staining and quantified by Visiopharm software. Total number and frequencies of tumor-infiltrating cytotoxic CD8a^+^ T cells were comparably augmented by any of our treatments (Fig. 6B; Supplementary Fig. S12A). The number of tumor-infiltrating GrB^+^ effector CD8a^+^ T cells was highest in mice treated with combinations of αPD-1 plus celecoxib or CAY10678 and somewhat increased in mice treated with αPD-1, celecoxib, or CAY10678 monotherapy (Fig. 6C). The percentage of GrB^+^CD8a^+^ T cells in total CD8a^+^ T cells was highest in mice treated with CA10678 monotherapy and combinations of αPD-1 plus celecoxib or CAY10678 (Supplementary Fig. S12B). In contrast, the number of tumor-infiltrating Tim3^+^ exhausted CD8a^+^ T cells was significantly lower in mice treated with single-agent CAY10678 or CAY10678 plus αPD-1 compared with any other treatments (Fig. 6D), and the percentage of Tim3^+^ exhausted CD8a^+^ T cells in total CD8a^+^ T cells was significantly suppressed by CAY10678-based treatments compared with celecoxib-based treatments although αPD-1 monotherapy also strongly suppressed the frequency (Supplementary Fig. S12C). The accumulation of CD8a^+^ DCs was induced by both celecoxib- and CAY10678-based treatments (Fig. 6E; Supplementary Fig. S12D). Neither celecoxib nor CAY10678 increased the number of tumor-infiltrating NK cells, as we observed in our KO models (Fig. 3F); however, intriguingly, tumor-infiltrating NK cells were significantly upregulated in mice treated with αPD-1–based regimens (Fig. 6F; Supplementary Fig. S12E). Celecoxib plus αPD-1 treatment was the only combination that enhanced the infiltration of B cells (Fig. 6G; Supplementary Fig. S12F). Finally, we assessed the presence of apoptotic tumor cells by evaluating the levels of cleaved caspase-3–positive cells (Fig. 6H), whose positivity was almost inversely related to the tumor weight (Fig. 5C).

## Discussion

The current study demonstrated that despite the common upstream/downstream enzymes responsible for the production of arachidonic acid metabolites, intrinsic mPGES-1 displays a specific function, distinct from that of COX-2, in modulating TIME in melanoma. Besides the recent intensive researches showing the roles of COX/PGE2 axis in evading tumor immunity (15–21), we, first time, uncovered the intriguing roles of mPGES-1 apart from mPGES-1/PGE2 axis. Genetic or pharmacologic inhibition of mPGES-1 was unique in that it could shift the TIME to favor cytotoxic CD8^+^ T-cell activation by excluding collagen production and Tim3^+^ exhausted phenotype. Accordingly, despite the inadequate inhibitory function of PGE2 synthesis, CAY10678 displayed noninferior therapeutic effects compared with celecoxib and was capable of boosting anti-PD-1 treatment without an increase of serious adverse events in a syngeneic mouse model.

Different arachidonic acid metabolite profiles induced by COX-2 and mPGES-1 inhibition have been proposed. Bergqvist and colleagues showed that compound III, a mPGES-1 inhibitor (same chemical structure with CAY10678), reduced PGE2 and increased PGF2α and TxB2 production while NS-398, a COX-2 inhibitor, blocked the production of them all (30). In addition, mPGES-1 inhibitor LY3023703, but not celecoxib, increased systemic PGI2 synthesis in humans (31). Similarly, genetic and pharmacologic inhibition of COX-2 and mPGES-1 conferred different metabolite profiles in our study. Inhibiting mPGES-1 may repartition PGH2 to the remaining prostaglandin synthases, resulting in increased PGD2, PGF2α, and PGI2 generation in a murine Braf^V600E^ melanoma model. Considering that each metabolite has a specific role in moderating tumor cells and neighboring immune cells (15–17, 32–36), it is likely that different profiles of prostaglandins released by tumor cells during genetic or pharmacologic depletion of COX-2 and mPGES-1 may lead to different phenotypes or functional properties of antitumor immune cells. In particular, PGD2 has been recognized as a tumor suppressor via the PGD2 receptor in colitis-associated colon cancer (33), and PGI2 is capable of recruiting CD4^+^ T cells in MHC class II–expressing murine lung cancer cells (34). Increased release of PGD2 and PGI2 by mPGES-1 inhibition may partially support the shift to favorable immune periphery in melanoma. In addition, previous studies have shown that each prostaglandin has positive or negative impact on collagen synthesis (37, 38). Therefore, it is of interest to determine whether the different balance of these prostaglandins is associated with the different collagen-related genes and collagen deposition.

Collagen is a predominant component of the extracellular matrix that may directly and indirectly influence the TIME to be advantageous for cancer development and progression. Collagen confers a physical barrier that makes it difficult for immune effectors to infiltrate the tumor microenvironment (39). In addition, recent studies have shown that collagen derives T-cell suppression through leukocyte-associated immunoglobulin-like receptor-1 (LAIR-1), an inhibitory collagen receptor expressed on immune cells (29, 40). David and colleagues discovered that collagen binds to LAIR-1 on T cells and activates the src homology region 2 domain–containing phosphatase 1 pathway, thereby inducing a T-cell exhaustion alternative to the PD-1 pathway in lung cancer (29). In our current study, we found that intrinsic mPGES-1 was associated with the expression of collagen-related genes, especially *col3a1*, *col4a1*, *col4a2*, and *col8a1*, and that genetic and pharmacologic depletion of mPGES-1 effectively decreased collagen deposition in the tumor microenvironment in a mouse model. TCGA-SKCM dataset corroborated the close correlation between mPGES-1 and these collagen mRNA levels. Moreover, frequencies of Tim3^+^ exhausted cytotoxic CD8a^+^ T cells were decreased by genetic depletion of mPGES-1 as well as by the treatment with an mPGES-1 inhibitor, suggesting collagen reduction by mPGES-1 inhibition may facilitate reinvigoration of exhausted T cells. The selection of ROIs at the same locations using serial sections for Masson's trichrome and mfIHC staining allowed us to assess how changes in collagen deposition affect T-cell exhaustion in this study (Supplementary Fig. S1B). In addition, the significant association between collagen and Tim3 gene expression (HAVCR2) in the dataset (Fig. 2E) also supports our suggestion. However, further investigation is warranted to clarify the mechanism by which mPGES-1 inhibition improves T-cell exhaustion and enhances anti-PD-1 therapy.

Building on previous research by our group and others (15, 25–27), we also accessed whether associations exist between DC, NK-cell, and B-cell infiltration into the tumor site and mPGES-1 and COX-2 expression in tumor cells. Compatible with findings from earlier studies (15, 25), genetic or pharmacologic depletion of COX-2 and mPGES-1 similarly enabled the recruitment of the classic CD8a^+^ DC subset, which is essential in initiating CD8^+^ T-cell priming and directing, into the tumor site by suppressing PGE2 production and provoking type I IFN-mediated chemokines; however, this subset was also trafficked by anti-PD-1 monotherapy, probably because of IFNγ produced by drug-activated T cells (41), and no synergistic effect of mPGES-1 and COX-2 inhibitors was observed. Although it has been noted that PGE2 was involved in suppressing NK-cell and B-cell activation and proliferation (21, 26, 27), neither the COX-2 nor mPGES-1 inhibitor was sufficient to recruit these cells to the tumor site in this study, indicating the insufficient stimulation of appropriate chemokines by these drugs. We did not examine the impact of the mPGES-1 inhibitor on the infiltration of immune-suppressive cell populations—including regulatory T cells, myeloid-derived suppressor cells, and tumor-associated macrophages—because our earlier study has already shown that genetic depletion of mPGES-1 did not suppress the number of tumor-infiltrating immune-suppressive cells (25).

The current study included several limitations. First, although we showed that genetic depletion of *ptges* and pharmacologic inhibition of mPGES-1 reduced collagen deposition and T-cell exhaustion, we did not prove the direct association between the decrease of collagen deposition and improvement of T-cell exhaustion. Investigation this association is of great importance to better understanding the specific roles of mPGES-1. Second, we evaluated only tumor cell–producing collagen deposition in TIME. Considering that collagen is also produced by peritumoral stroma, it is conceivable that genetic depletion of *ptges* or pharmacologic inhibition of mPGES-1 indirectly decrease collagen deposition by regulating the function of peritumoral stroma. Third, we suggested in this study that intrinsic mPGES-1 expression in melanoma was linked with several collagen genes by RNA-seq data analysis using a mouse melanoma cell line and the analysis of TCGA-SKCM dataset. However, the data of TCGA-SKCM dataset should be interpreted cautiously as human melanoma cells included not only tumor cells but also peritumor cells including stromal and immune cells, and these cells may contribute to the expression of collagen genes.

In conclusion, intrinsic mPGES-1 uniquely functions as an inducer of collagen deposition and a modulator of cytotoxic T-cell exhaustion; and specific pharmacologic inhibition of mPGES-1 may hold therapeutic promise as a safer alternative to COX-2 inhibitors for improving immune checkpoint–based therapies in melanoma. However, to date, no current mPGES-1 inhibitor has been approved for human use, with early candidates exhibiting unique noncardiac toxicities (31, 42). Therefore, further development of mPGES-1 inhibitors for human use is encouraged and is expected to broaden adjuvant treatment options for immunotherapy in melanoma.

## Supplementary Material

Supp Table S1 Supplementary Table 1

Supp Table S2 Supplementary Table 2

Supp Figure S1 Figure S1 shows the selection of ROIs in tumors

Supp Figure S2 Figure S2 summarizes the calculation details of collagen positivity

Supp Figure S3 Figure S3 shows mfIHC staining details

Supp Figure S4 Figure S4 explains quantitative analysis of tumor-infiltrating immune cells

Supp Figure S5 Figure S5 details the conditions of RNA sequencing analyses

Supp Figure S6 Figure S6 shows the analysis of collagen mRNA levels in ptgs1-KO, ptgs2-KO, and ptges-KO cell lines in S6A to H; qRT-PCR results in S6I; and Venn diagram showing the common and significant collagen genes in S6J

Supp Figure S7 Figure S7 shows the correlation between PTGES mRNA level and collagen-related genes in a cohort of patients with advanced melanoma

Supp Figure S8 Figure S8 shows morphological differences between ptgs1-, ptgs2-, and ptges-KO murine BrafV600E melanoma cells by using phase-contrast microscopy

Supp Figure S9 Figure S9 shows frequencies of tumor-infiltrating immune cells in tumors derived from ptgs2-KO and ptges-KO murine BrafV600E melanoma cells

Suppl Figure S10 Figure S10 details the distribution of tumor-infiltrating immune cells in tumors derived from ptgs2-KO and ptges-KO murine BrafV600E melanoma cells

Suppl Figure S11 Figure S11 shows the effect of CAY10678 on the production of arachidonic acid metabolites from murine BrafV600E melanoma cells

Suppl Figure S12 Figure S12 shows the Frequencies of tumor-infiltrating immune cells in tumors treated with celecoxib or CAY10678 as monotherapy or combined with and PD-1 blockade
