# Supplementary material for: Inhibition of Microsomal Prostaglandin E2 Synthase Reduces Collagen Deposition in Melanoma Tumors and May Improve Immunotherapy Efficacy by Reducing T-cell Exhaustion
Source: Cancer Res Commun. 2023 Jul 31;3(7):1397–408. doi: 10.1158/2767-9764.CRC-23-0210 (PMC10389052; doi:10.1158/2767-9764.CRC-23-0210)
Supplement: Supp Table S1 — Supplementary Table 1 [file crc-23-0210-s01.pdf]

Supplementary Table 1. Information of antibodies (Abs) and paired Opal-fluorophores used for mfIHC.

Panel	Staining order	Ab	Dilution	Clone	Catalog No.	Vendor	Fluorophore	Dilution
Panel 1	1	CD3	1:200	Polyclonal	ab5690	Abcam	Opal Polaris 480	1:250
	2	CD8a	1:100	4sm15	14-0808-82	eBioscience	Opal 620	1:100
	3	Tim3	1:200	D3M9R	83882s	Cell Signaling	Opal 690	1:100
	4	Granzyme B	1:200	Polyclonal	NB100-684	Novus Biologicals	Opal 520	1:150
	5	CD45	1:500	30-F11	70-0451	Tonbo Biosciences	Opal Polaris780	1:25
Panel 2	1	CD8a	1:100	4sm15	14-0808-82	eBioscience	Opal 620	1:100
	2	CD11b	1:750	Polyclonal	NB110-89474	Novus Biologicals	Opal 570	1:100
	3	CD11c	1:300	D1V9Y	97585s	Cell Signaling	Opal 520	1:150
	4	CD45	1:500	30-F11	70-0451	Tonbo Biosciences	Opal Polaris780	1:25
Panel 3	1	NK1.1	1:500	PK136	NB100-77528	Novus Biologicals	Opal Polaris480	1:250
	2	CD19	1:200	Polyclonal	HS-439 003	Synaptic Systems	Opal 620	1:100
	3	CD45	1:500	30-F11	70-0451	Tonbo Biosciences	Opal 570	1:100
	4	Gp100	1:200	EP4863(2)	ab137078	Abcam	Opal 690	1:100
	5	Cleaved caspase 3	1:200	5A1E	9664s	Cell Signaling	Opal 520	1:100
Panel 4	1	PD-1	1:200	D7D5M	84651	Cell Signaling	Opal 690	1:100
	2	LAG3	1:250	Polyclonal	AF3328	R&D Systems	Opal 570	1:100
	3	Tim3	1:200	D3M9R	83882s	Cell Signaling	Opal 520	1:150
	4	CD8a	1:100	4sm15	14-0808-82	eBioscience	Opal Polaris 480	1:250

5	IFN-γ	1:500	Polyclonal	BS-0480R	Bioss	Opal 620	1:100
6	CD45	1:500	30-F11	70-0451	Tonbo Biosciences	Opal Polaris780	1:25
