# Supplementary material for: Inhibition of Microsomal Prostaglandin E2 Synthase Reduces Collagen Deposition in Melanoma Tumors and May Improve Immunotherapy Efficacy by Reducing T-cell Exhaustion
Source: Cancer Res Commun. 2023 Jul 31;3(7):1397–408. doi: 10.1158/2767-9764.CRC-23-0210 (PMC10389052; doi:10.1158/2767-9764.CRC-23-0210)
Supplement: Supp Table S2 — Supplementary Table 2 [file crc-23-0210-s02.pdf]

Supplementary Table 2. Identification of immune and tumor cell lineage markers.

Cell lineage	Marker
Cytotoxic $CD8a^{+}$ T cell	$CD45^{+}CD3^{+}CD8a^{+}$
Granzyme B $(GrB)^{+}$ effector $CD8a^{+}$ T cell	$CD45^{+}CD3^{+}CD8a^{+}GrB^{+}$
IFN-γ^{+} effector $CD8a^{+}$ T cell	$CD45^{+}CD8a^{+}$IFN-γ^{+}
IFN-γ^{+} $Tim3^{-}$ effector $CD8a^{+}$ T cell	$CD45^{+}CD8a^{+}$IFN-$\gamma^{+}Tim3^{-}$
$Tim3^{+}$ exhausted $CD8a^{+}$ T cell	$CD45^{+}CD3^{+}CD8a^{+}Tim3^{+}$
$Tim3^{+}$ PD-1^{+} $LAG3^{+}$ exhausted $CD8a^{+}$ T cell	$CD45^{+}CD8a^{+}Tim3^{+}$PD-$1^{+}LAG3^{+}$
$CD8a^{+}$ dendritic cell (DC)	$CD45^{+}CD11b^{-}CD11c^{+}CD8a^{+}$
Natural killer (NK) cell	$CD45^{+}NK1.1^{+}$
B cell	$CD45^{+}CD19^{+}$
Cleaved caspase3 $(CC3)^{+}$ tumor cell	$gp100^{+}CC3^{+}$
