# Supplementary material for: Inhibition of Microsomal Prostaglandin E2 Synthase Reduces Collagen Deposition in Melanoma Tumors and May Improve Immunotherapy Efficacy by Reducing T-cell Exhaustion
Source: Cancer Res Commun. 2023 Jul 31;3(7):1397–408. doi: 10.1158/2767-9764.CRC-23-0210 (PMC10389052; doi:10.1158/2767-9764.CRC-23-0210)
Supplement: Supp Figure S1 — Figure S1 shows the selection of ROIs in tumors [file crc-23-0210-s03.pdf]

Supplementary Figure S1.

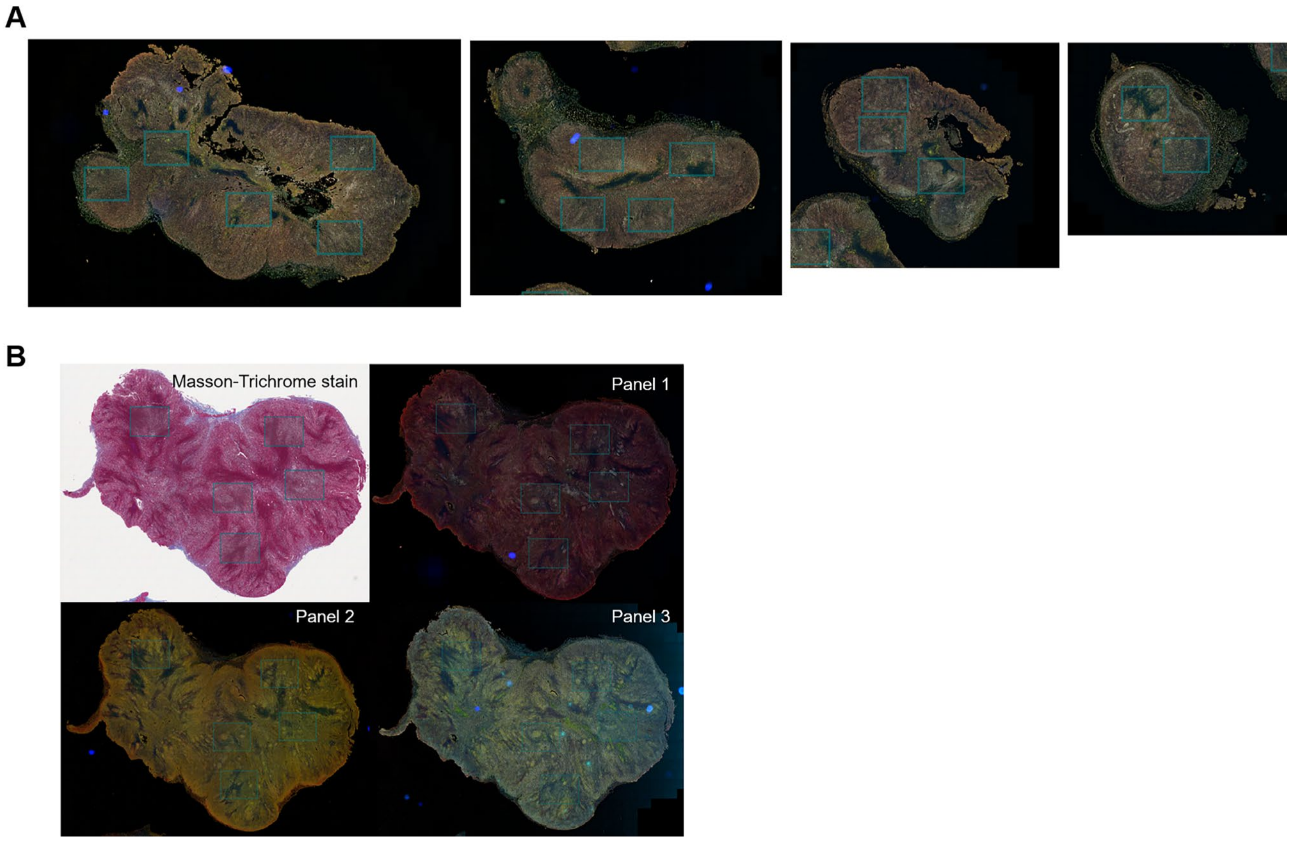

Supplementary Figure S1. Selection of ROIs from tumors.

A, Representative images of ROIs (green rectangles) selected from tumors. The number of ROIs selected from each tumor was dependent on tumor size (2-5 ROIs). Each ROI is 1.1×1.5 mm.

B, Representative images of ROI locations (green rectangles) for Masson’s trichrome stained and mfIHC stained slides in each tumor sample.
