# Supplementary material for: Inhibition of Microsomal Prostaglandin E2 Synthase Reduces Collagen Deposition in Melanoma Tumors and May Improve Immunotherapy Efficacy by Reducing T-cell Exhaustion
Source: Cancer Res Commun. 2023 Jul 31;3(7):1397–408. doi: 10.1158/2767-9764.CRC-23-0210 (PMC10389052; doi:10.1158/2767-9764.CRC-23-0210)
Supplement: Supp Figure S2 — Figure S2 summarizes the calculation details of collagen positivity [file crc-23-0210-s04.pdf]

Supplementary Figure S2.

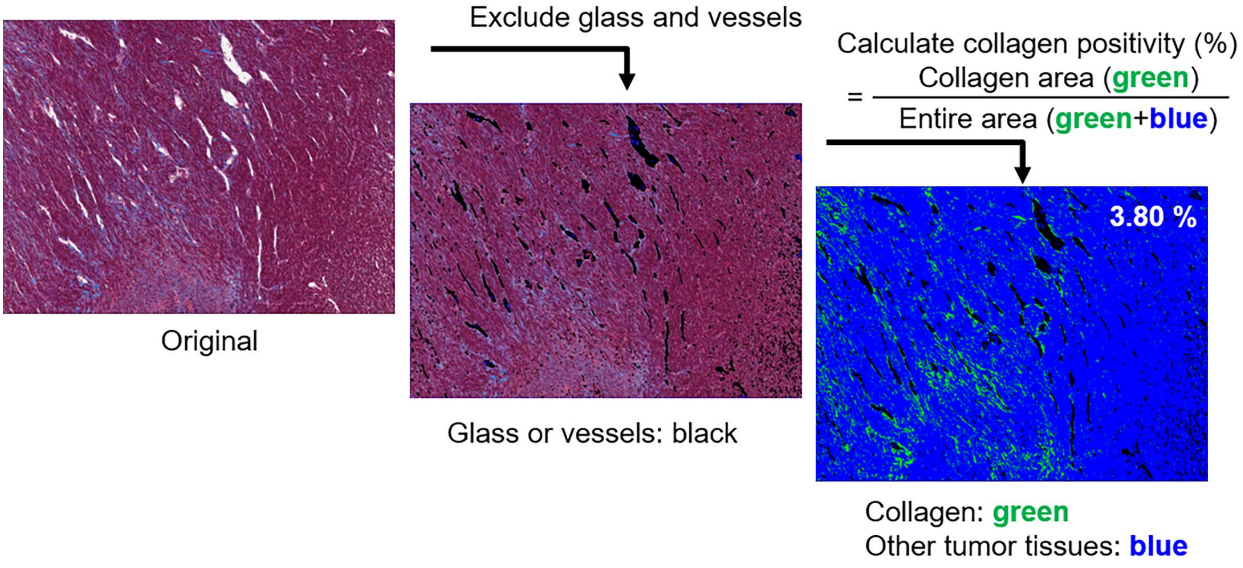

Supplementary Figure S2. Calculation of collagen positivity.

Background glass area and vessel region were excluded from analysis for each ROI. Next, the collagen-positive area was automatically identified by Visiopharm. Collagen positivity was calculated by dividing collagen-positive area by the entire area.
