# Supplementary material for: Inhibition of Microsomal Prostaglandin E2 Synthase Reduces Collagen Deposition in Melanoma Tumors and May Improve Immunotherapy Efficacy by Reducing T-cell Exhaustion
Source: Cancer Res Commun. 2023 Jul 31;3(7):1397–408. doi: 10.1158/2767-9764.CRC-23-0210 (PMC10389052; doi:10.1158/2767-9764.CRC-23-0210)
Supplement: Supp Figure S3 — Figure S3 shows mfIHC staining details [file crc-23-0210-s05.pdf]

Supplementary Figure S3.

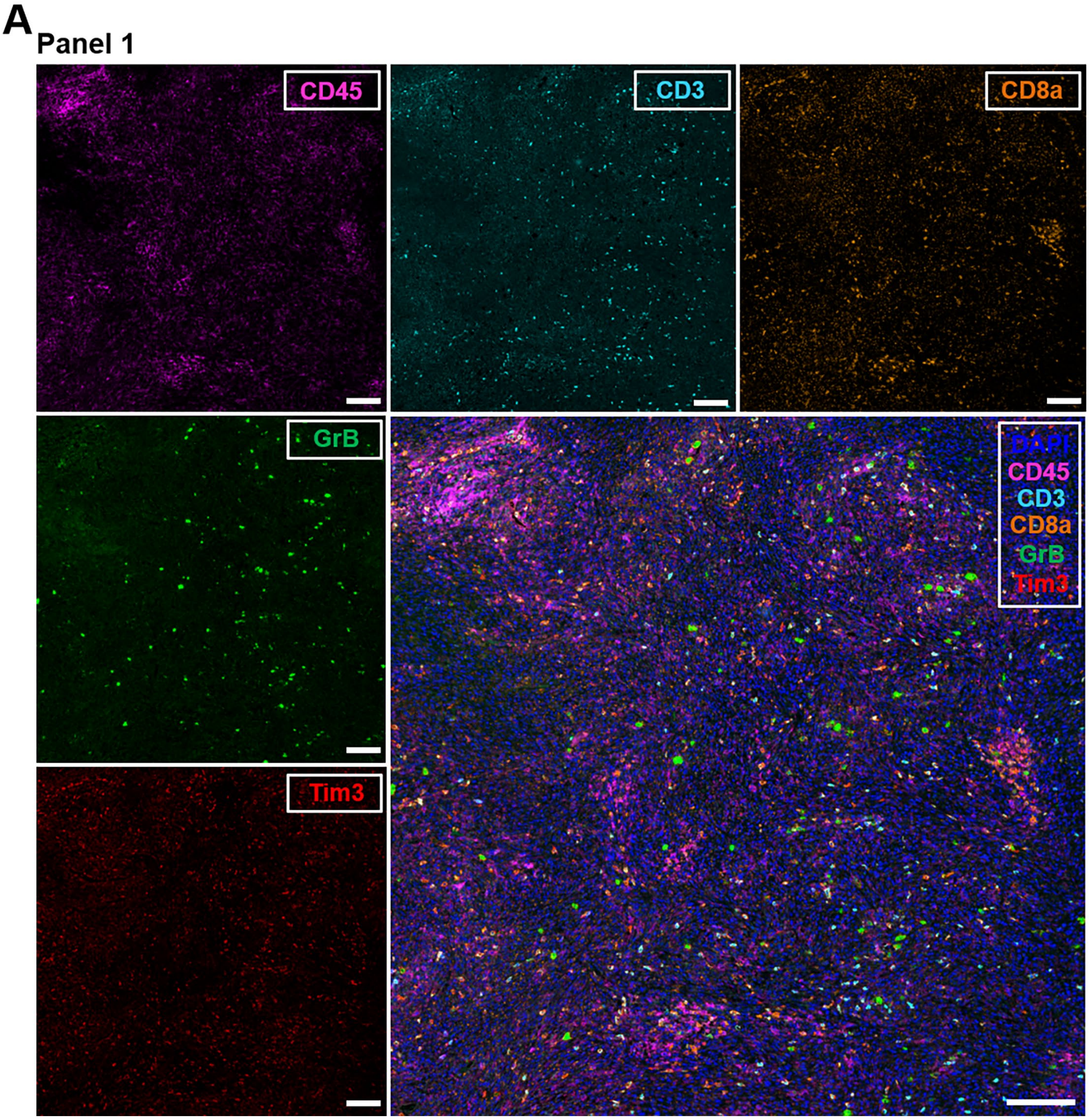

B Panel 2

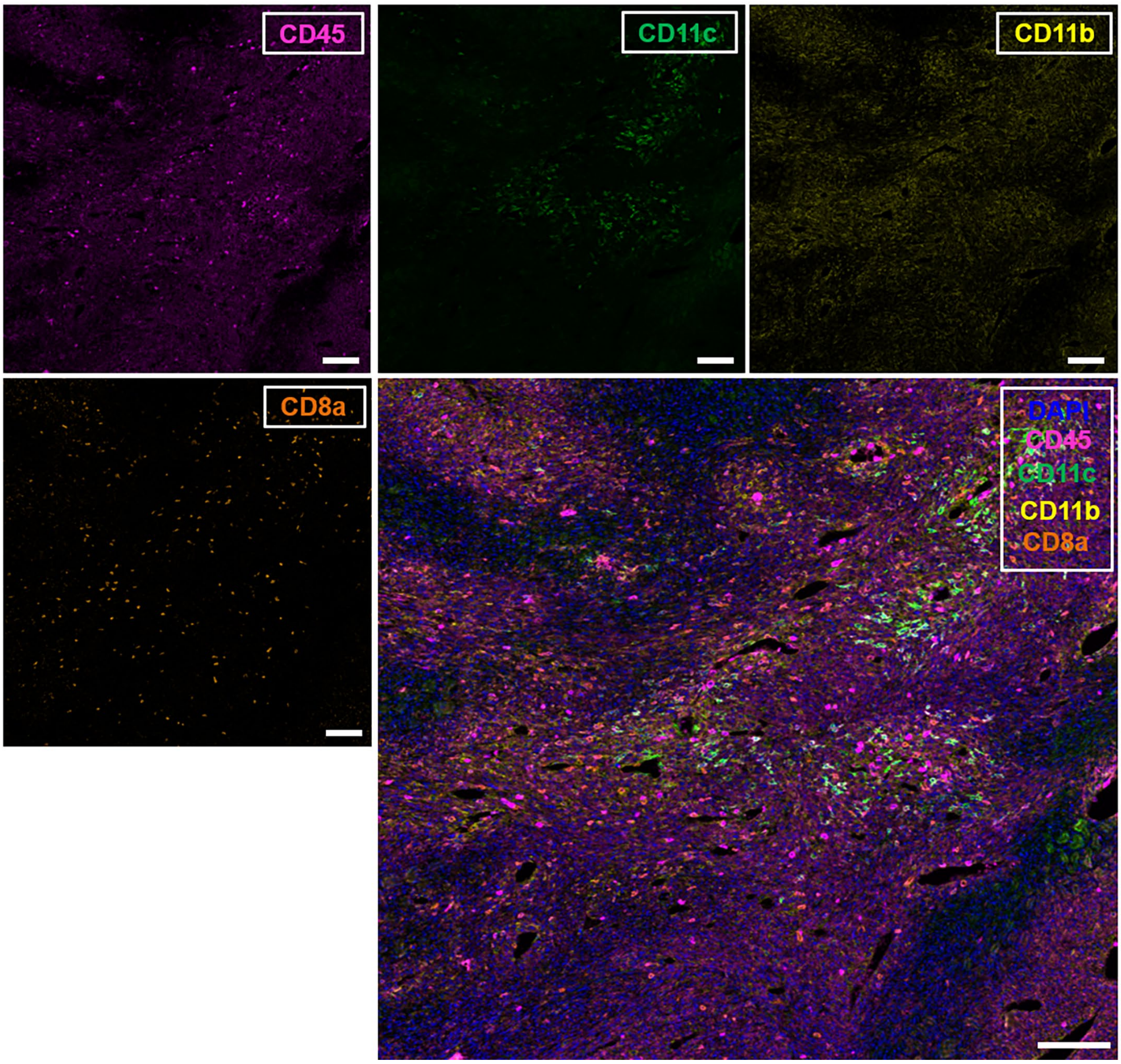

C

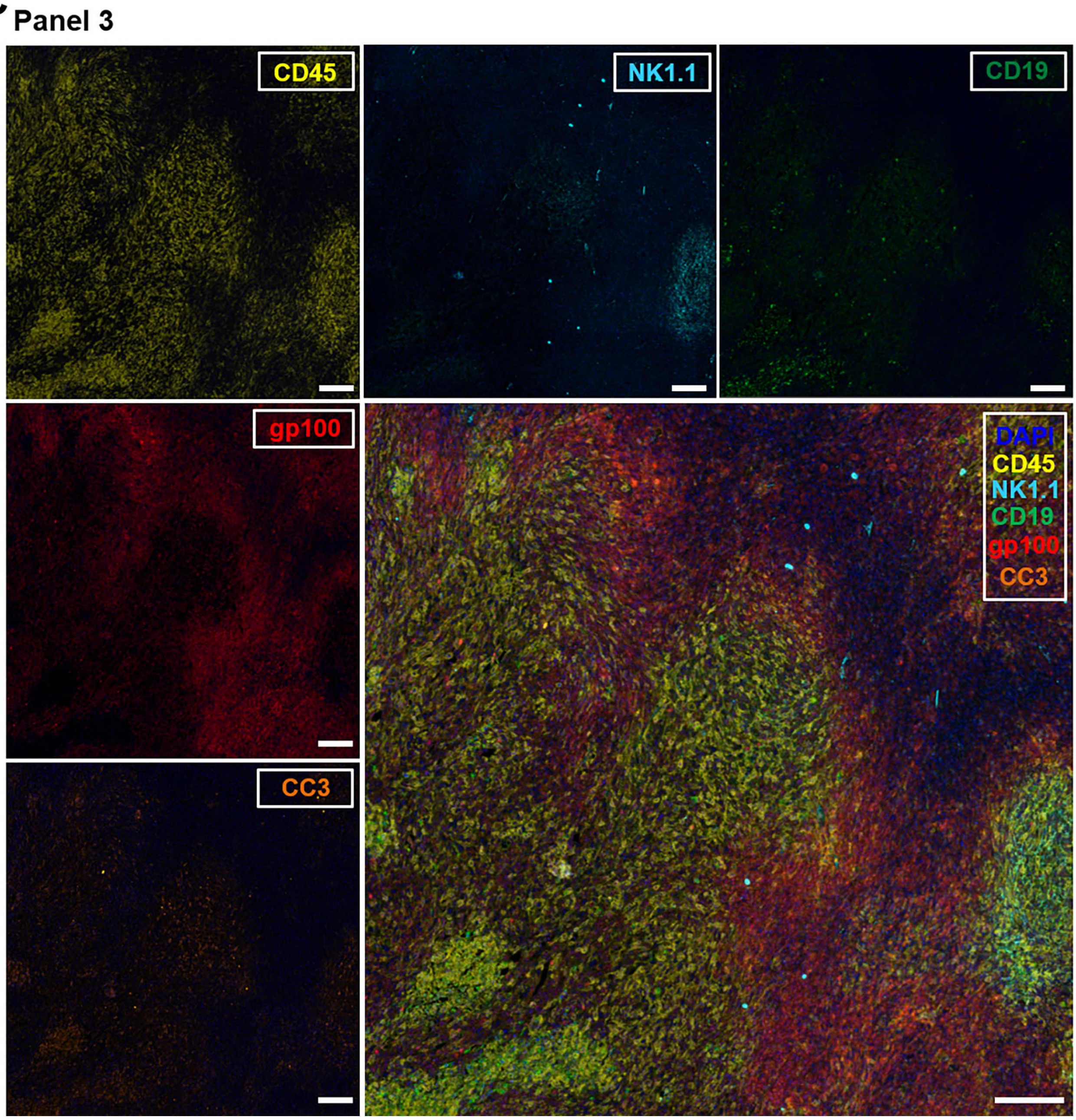

Panel 3
CD45
NK1.1
CD19
gp100
CC3
DAPI
CD45
NK1.1
CD19
gp100
CC3

D

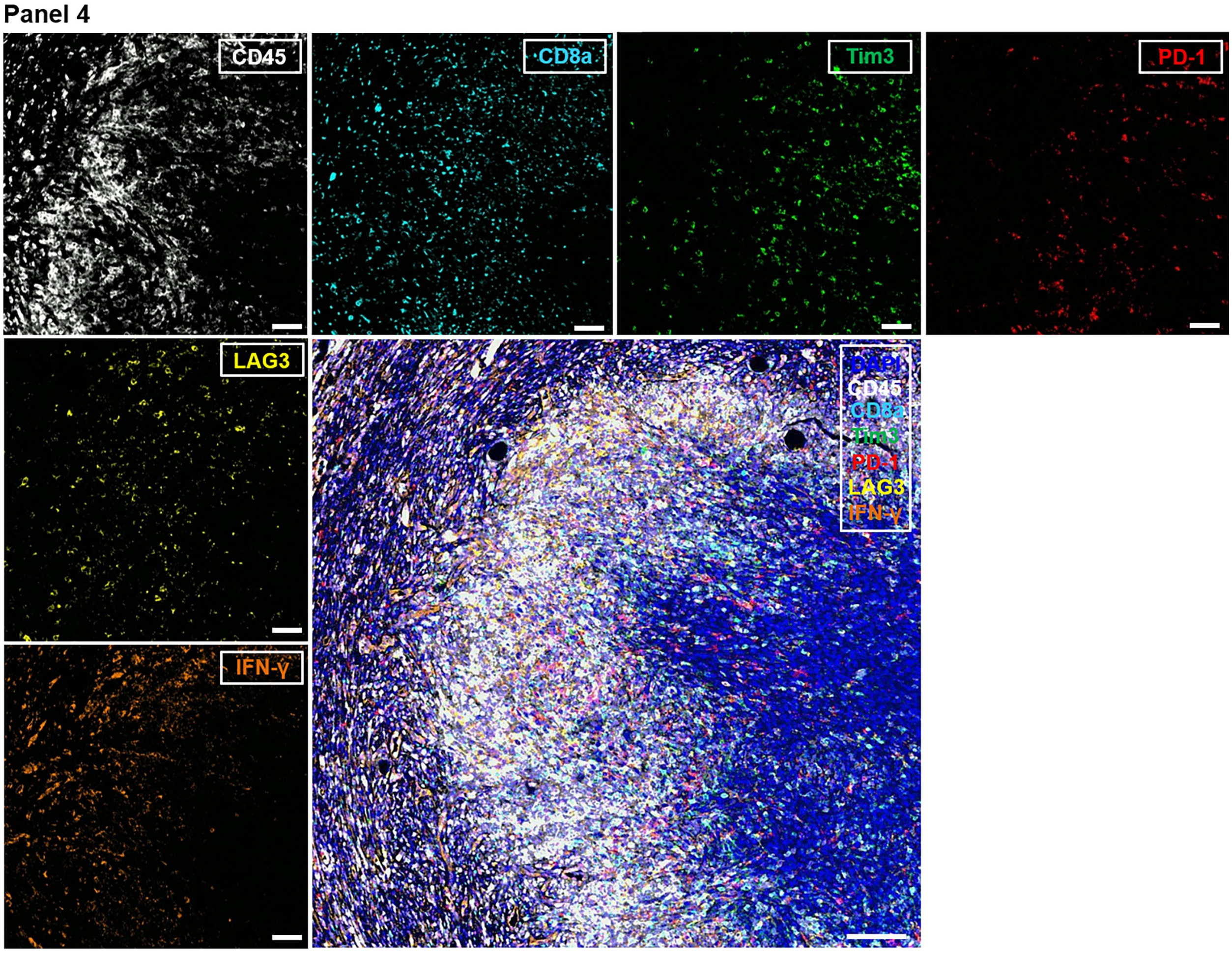

Supplementary Figure S3. mfIHC staining for three panels.

A, Representative images of multiplex fluorescent immunohistochemistry staining for CD45, CD3, CD8a, granzyme B (GrB), Tim3, and DAPI in Panel 1. Scale bar = 100µm. **B,** Representative images of multiplex fluorescent immunohistochemistry staining for CD45, CD11c, CD11b, CD8a, and DAPI in Panel 2. Scale bar = 100µm. **C,** Representative images of multiplex fluorescent immunohistochemistry staining for CD45, NK1.1, CD19, gp100, CC3, and DAPI in Panel 3. Scale bar = 100 µm. **D,** Representative images of multiplex fluorescent immunohistochemistry staining for CD45, CD8a, Tim3, PD-1, LAG3, IFN-γ, and DAPI in Panel 4. Scale bar = 100 µm.
