# Supplementary material for: Inhibition of Microsomal Prostaglandin E2 Synthase Reduces Collagen Deposition in Melanoma Tumors and May Improve Immunotherapy Efficacy by Reducing T-cell Exhaustion
Source: Cancer Res Commun. 2023 Jul 31;3(7):1397–408. doi: 10.1158/2767-9764.CRC-23-0210 (PMC10389052; doi:10.1158/2767-9764.CRC-23-0210)
Supplement: Supp Figure S4 — Figure S4 explains quantitative analysis of tumor-infiltrating immune cells [file crc-23-0210-s06.pdf]

Supplementary Figure S4.

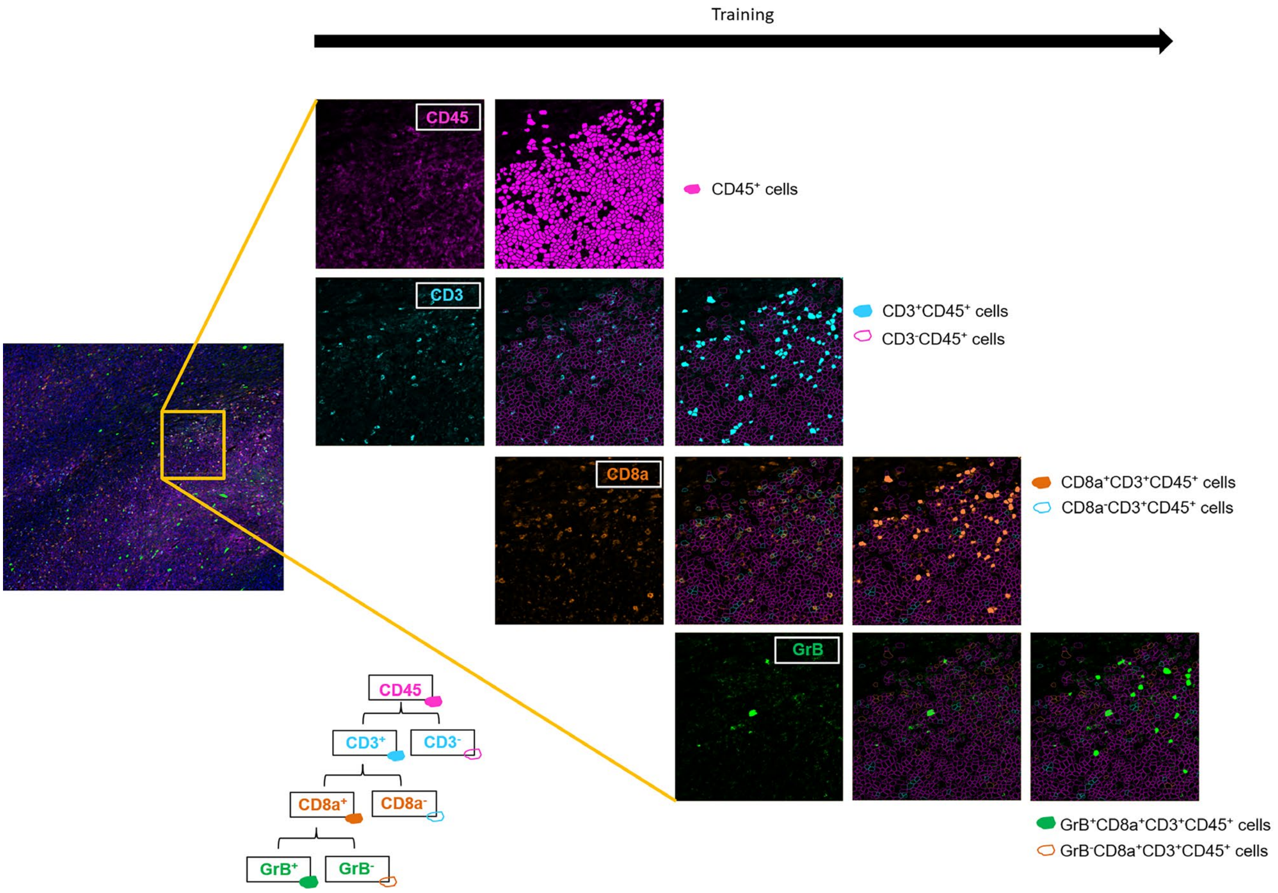

Supplementary Figure S4. Quantitative analysis of tumor-infiltrating immune cells. Representative images of multiplex fluorescent immunohistochemistry staining for CD45, CD3, CD8a, and GrB. The signal positivity threshold for each marker was manually optimized on a one-by-one basis, and the number of signal-positive or -negative cells was automatically counted in a step-by-step manner using Visiopharm.
