# Supplementary material for: Inhibition of Microsomal Prostaglandin E2 Synthase Reduces Collagen Deposition in Melanoma Tumors and May Improve Immunotherapy Efficacy by Reducing T-cell Exhaustion
Source: Cancer Res Commun. 2023 Jul 31;3(7):1397–408. doi: 10.1158/2767-9764.CRC-23-0210 (PMC10389052; doi:10.1158/2767-9764.CRC-23-0210)
Supplement: Supp Figure S5 — Figure S5 details the conditions of RNA sequencing analyses [file crc-23-0210-s07.pdf]

Supplementary Figure S5.

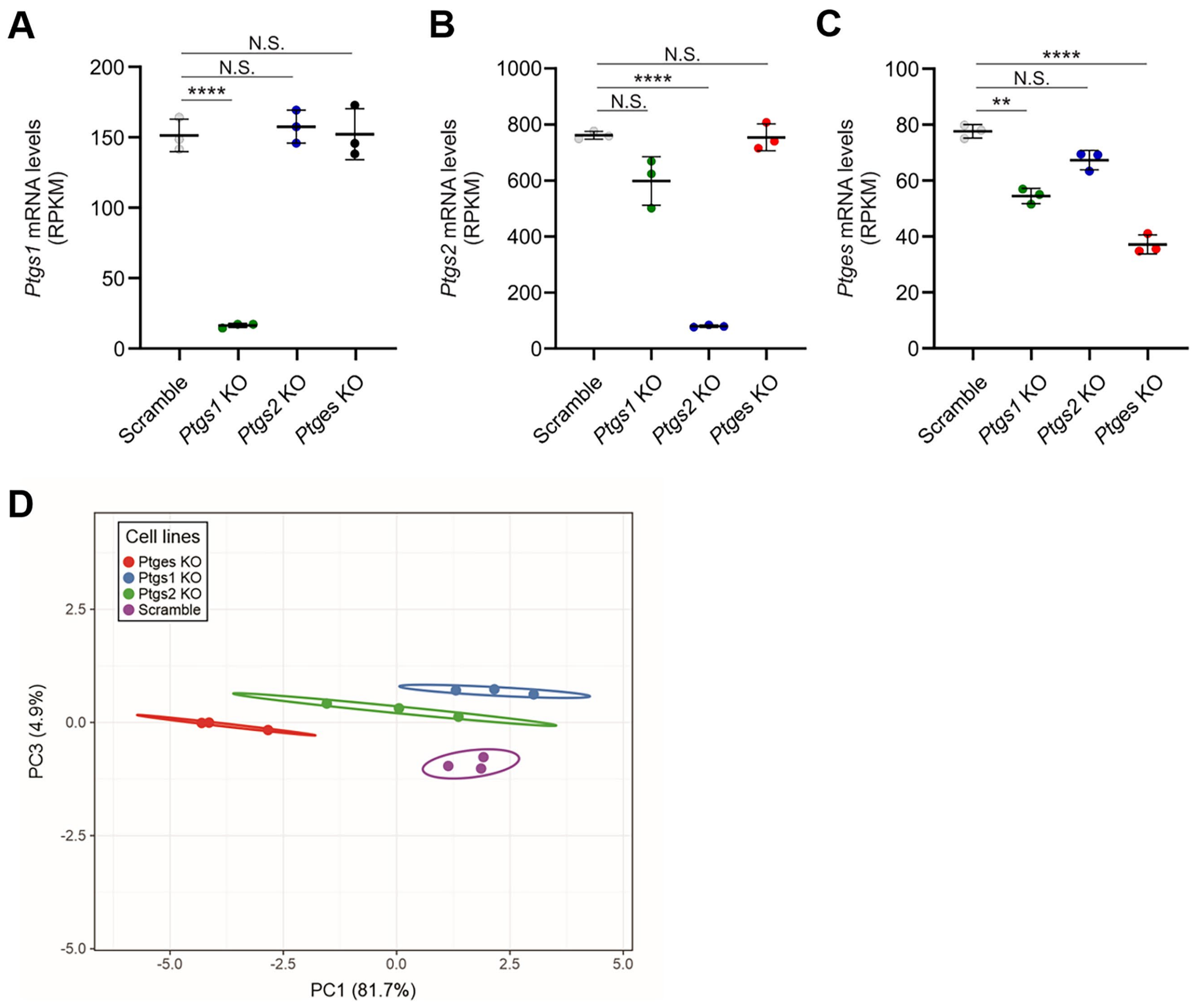

Supplementary Figure S5. Conditions of RNA sequencing analyses.

A-C, *Ptgs1*, *ptgs2*, and *ptges* mRNA levels in three samples of scramble, *ptgs1*-KO, *ptgs2*-KO, and *ptges*-KO murine Braf^{V600E} melanoma cells: *ptgs1* (A), *ptgs2* (B), *ptges* (C). Statistical differences between groups were compared using one-way ANOVA and Tukey's multiple comparisons test.

D, PCA plot comparing scramble and *ptgs1*-KO, *ptgs2*-KO, and *ptges*-KO murine Braf^{V600E} melanoma cell lines that were analyzed by RNA-Seq. Only eight collagen genes with an average count greater than 10 in RNA-Seq analysis were included in the analysis. Shown are the principal components (PC) 1 and 3. Graph values represent mean ± SD. $**p < 0.01$, $****p < 0.0001$. N.S., not statistically significant.
