# Supplementary material for: Inhibition of Microsomal Prostaglandin E2 Synthase Reduces Collagen Deposition in Melanoma Tumors and May Improve Immunotherapy Efficacy by Reducing T-cell Exhaustion
Source: Cancer Res Commun. 2023 Jul 31;3(7):1397–408. doi: 10.1158/2767-9764.CRC-23-0210 (PMC10389052; doi:10.1158/2767-9764.CRC-23-0210)
Supplement: Supp Figure S6 — Figure S6 shows the analysis of collagen mRNA levels in ptgs1-KO, ptgs2-KO, and ptges-KO cell lines in S6A to H; qRT-PCR results in S6I; and Venn diagram showing the common and significant collagen genes in S6J [file crc-23-0210-s08.pdf]

Supplementary Figure S6.

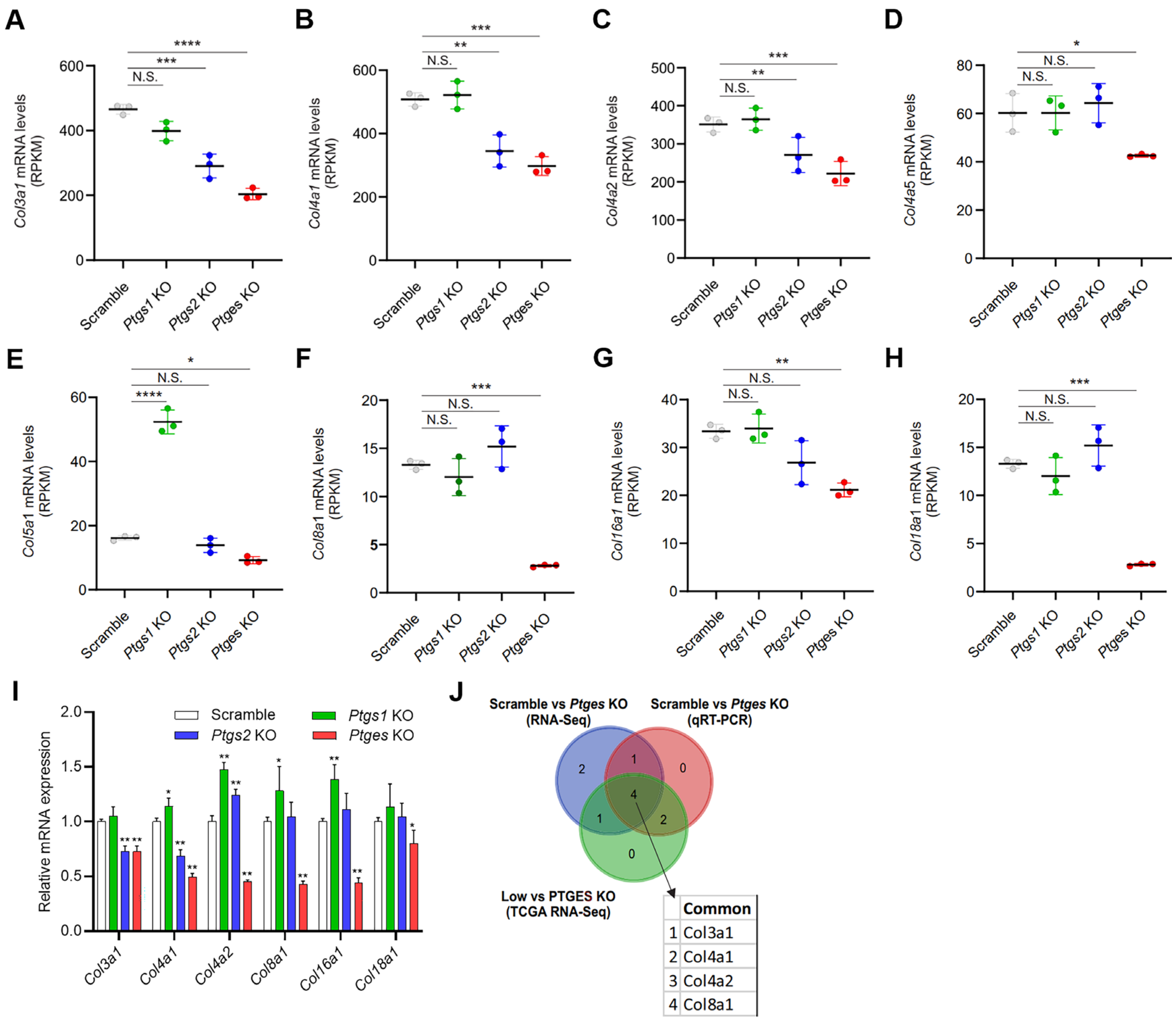

Supplementary Figure S6. Analysis of collagen mRNA levels in *ptgs1*-KO, *ptgs2*-KO, and *ptges*-KO cell lines.

A-H, The mRNA levels of eight collagen-related genes were compared between scramble and *ptgs1*-KO, *ptgs2*-KO, and *ptges*-KO cells: *col3a1* (A), *col4a1* (B), *col4a2* (C), *col4a5* (D), *col5a1* (E), *col8a1* (F), *col16a1* (G), and *col18a1* (H). **I,** qRT-PCR analyses for *col3a1*, *col4a1*, *col4a2*, *col8a1*, *col16a1*, and *col18a1* genes in scramble, *ptgs1*-KO, *ptgs2*-KO, and *ptges*-KO cells. Bar graphs show the fold-change relative to mRNA levels of scramble control for each gene (n = 4). **J,** Venn diagram showing the common and significant collagen genes in *ptges*-KO vs scramble cells (scramble vs *ptges*-KO in RNA-Seq and qRT-PCR) and TCGA-SKCM low-*PTGES* vs high-*PTGES* groups. Data represent the mean ± SD. Statistical differences between groups were compared using One-way ANOVA and Tukey's multiple comparisons test. * $p < 0.05$, ** $p < 0.01$, *** $p < 0.001$, **** $p < 0.0001$. N.S., not statistically significant.
