# Supplementary material for: Inhibition of Microsomal Prostaglandin E2 Synthase Reduces Collagen Deposition in Melanoma Tumors and May Improve Immunotherapy Efficacy by Reducing T-cell Exhaustion
Source: Cancer Res Commun. 2023 Jul 31;3(7):1397–408. doi: 10.1158/2767-9764.CRC-23-0210 (PMC10389052; doi:10.1158/2767-9764.CRC-23-0210)
Supplement: Supp Figure S7 — Figure S7 shows the correlation between PTGES mRNA level and collagen-related genes in a cohort of patients with advanced melanoma [file crc-23-0210-s09.pdf]

**Supplementary Figure S7.**

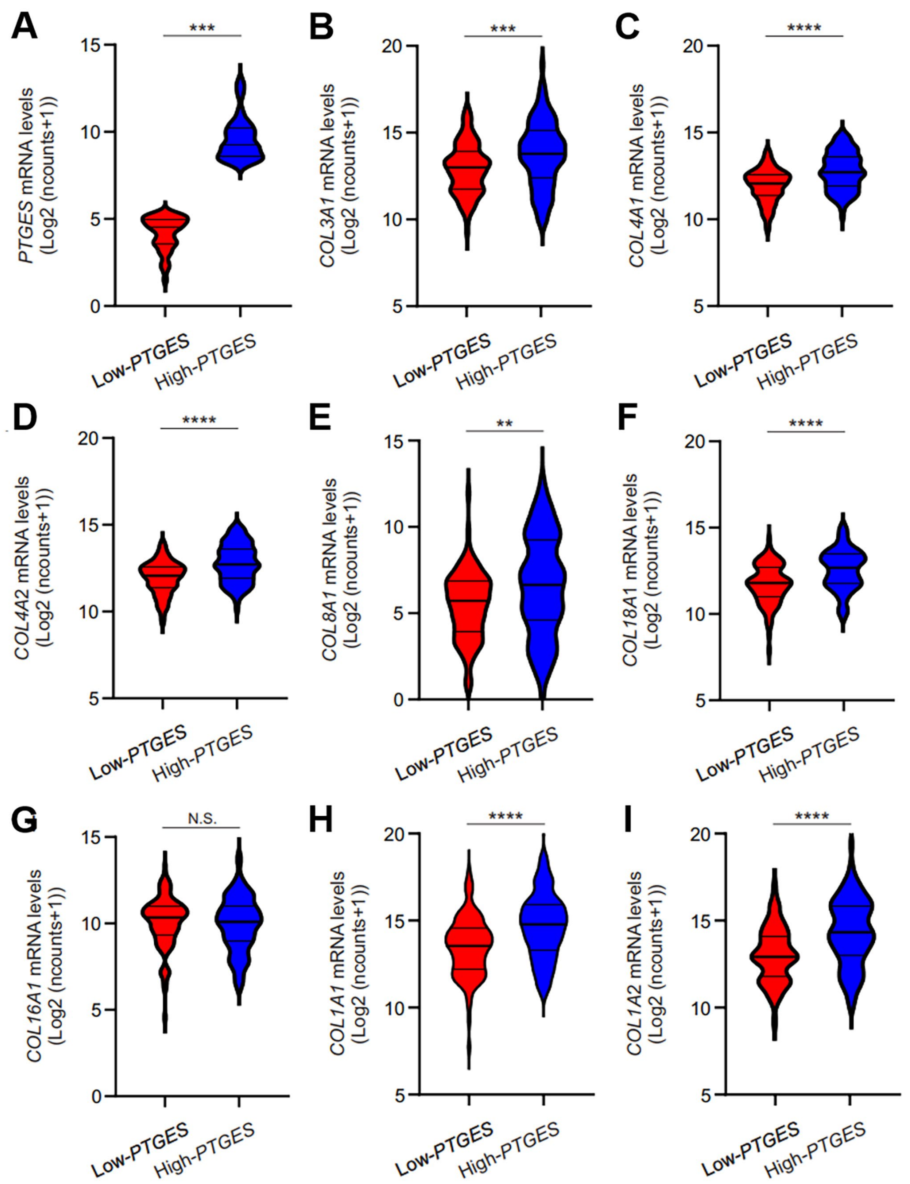


**Supplementary Figure S7. Correlation between *PTGES* mRNA level and collagen-related genes in a cohort of patients with advanced melanoma.**

**A,** *PTGES* mRNA levels in patients with high and low *PTGES*. **B-I,** Violin plots depicting the mRNA levels of 8 collagen-related genes in patients with high and low *PTGES*: *COL3A1* (B), *COL4A1* (C), *COL4A2* (D), *COL8A1* (E), *COL18A1* (F), *COL16A1* (G), *COL1A1* (H), and *COL1A2* (I). Stage III-IV melanoma patients extracted from the TCGA-SKCM dataset were divided into four quartiles, and patients with the highest *PTGES* mRNA levels (n=92) were compared with the lowest levels (n=92) to determine the levels of collagen-related genes. Statistical differences between groups were compared using Student's *t*-test. Data represent the mean ± SD.**$p < 0.01$, ***$p < 0.001$, ****$p < 0.0001$. N.S., not statistically significant.
