# Supplementary material for: Inhibition of Microsomal Prostaglandin E2 Synthase Reduces Collagen Deposition in Melanoma Tumors and May Improve Immunotherapy Efficacy by Reducing T-cell Exhaustion
Source: Cancer Res Commun. 2023 Jul 31;3(7):1397–408. doi: 10.1158/2767-9764.CRC-23-0210 (PMC10389052; doi:10.1158/2767-9764.CRC-23-0210)
Supplement: Supp Figure S8 — Figure S8 shows morphological differences between ptgs1-, ptgs2-, and ptges-KO murine BrafV600E melanoma cells by using phase-contrast microscopy [file crc-23-0210-s10.pdf]

Supplementary Figure S8.

A

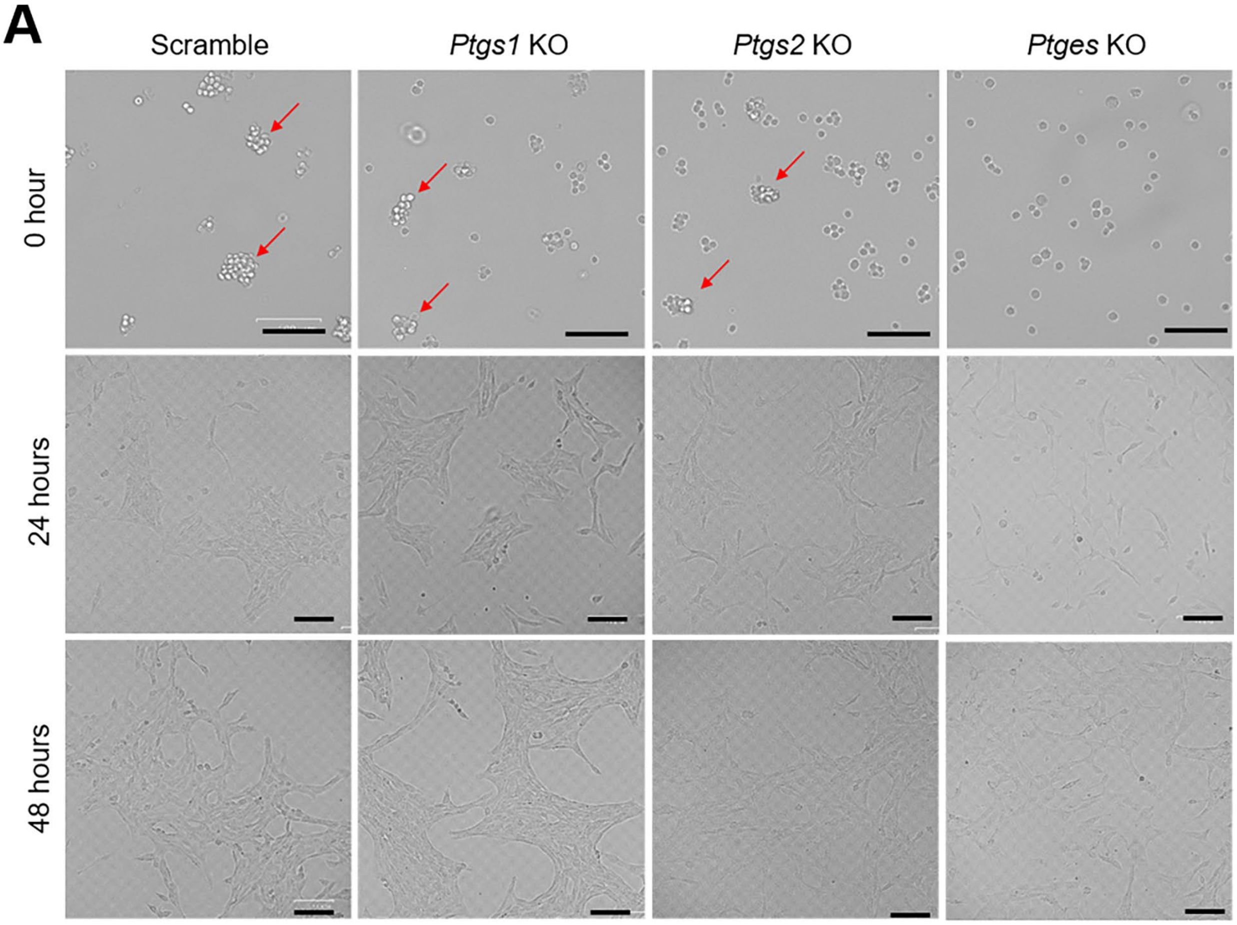

B

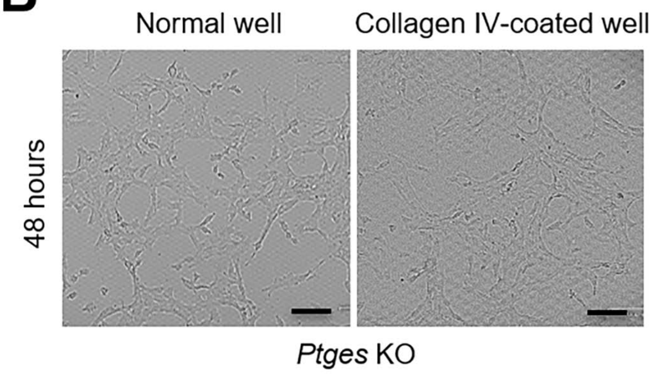

Supplementary Figure S8. Morphological differences between *ptgs1*-, *ptgs2*-, and *ptges*-KO murine Braf^{V600E} melanoma cells.

A, Representative images of scramble, *ptgs1*-KO, *ptgs2*-KO, and *ptges*-KO cells after 0, 24, and 48 hours culture using phase-contrast microscopy. Red arrows indicate the aggregated cells. Scale bar = 100 μm. **B,** Representative images of *ptges*-KO cells after 48 hours culture on normal well (left) and collagen IV-coated well (right) using phase-contrast microscopy. Scale bar = 100 μm.
