# Supplementary material for: Inhibition of Microsomal Prostaglandin E2 Synthase Reduces Collagen Deposition in Melanoma Tumors and May Improve Immunotherapy Efficacy by Reducing T-cell Exhaustion
Source: Cancer Res Commun. 2023 Jul 31;3(7):1397–408. doi: 10.1158/2767-9764.CRC-23-0210 (PMC10389052; doi:10.1158/2767-9764.CRC-23-0210)
Supplement: Suppl Figure S10 — Figure S10 details the distribution of tumor-infiltrating immune cells in tumors derived from ptgs2-KO and ptges-KO murine BrafV600E melanoma cells [file crc-23-0210-s12.pdf]

Supplementary Figure S10.

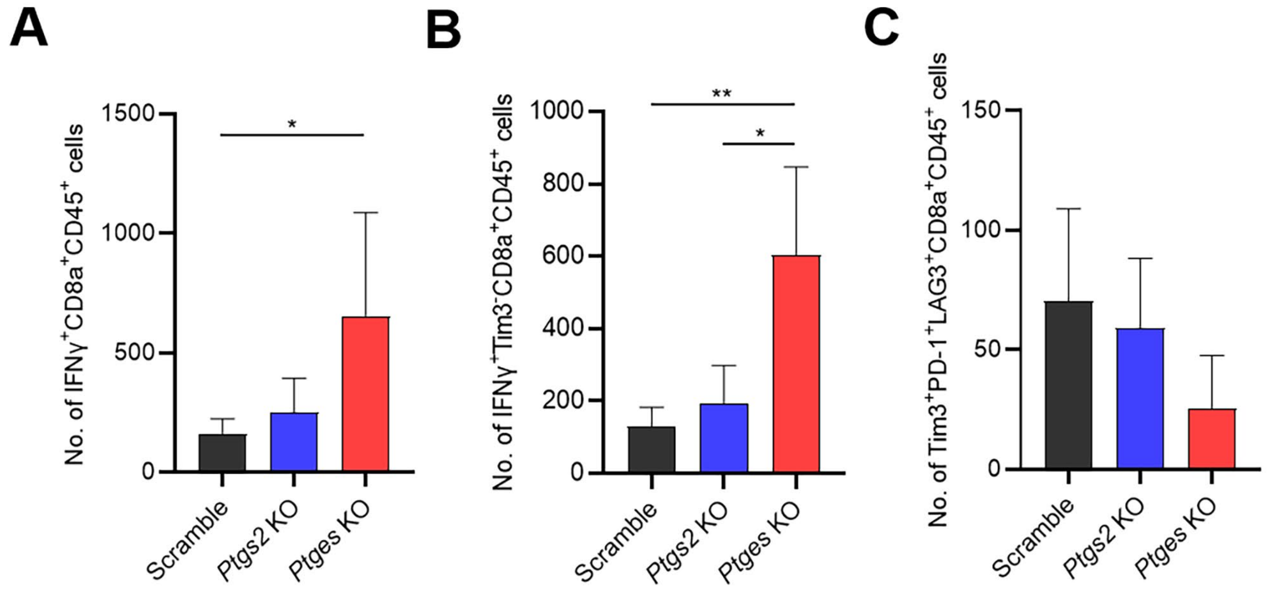

Supplementary Figure S10. Distribution of tumor-infiltrating immune cells in tumors derived from *ptgs2*-KO and *ptges*-KO murine Braf^{V600E} melanoma cells.

A-C, Distribution of tumor-infiltrating immune cells was compared between tumors from scramble, *ptgs2*-KO, and *ptges*-KO cells. The number of tumor-infiltrating immune cells was automatically calculated using Visiopharm software. Shown are the numbers of tumor-infiltrating IFN-γ^+ effector CD8a$^+$ T cells (A), tumor-infiltrating IFN-γ^+Tim3$^-$ effector CD8a$^+$ T cells (B), tumor-infiltrating Tim3$^+$PD-1$^+$LAG3$^+$ exhausted CD8a$^+$ T cells (C). Graph values represent mean ± SD. Significance in difference between two groups was determined by Student *t*-test. $*p < 0.05$, $**p < 0.01$.
