# Supplementary material for: Inhibition of Microsomal Prostaglandin E2 Synthase Reduces Collagen Deposition in Melanoma Tumors and May Improve Immunotherapy Efficacy by Reducing T-cell Exhaustion
Source: Cancer Res Commun. 2023 Jul 31;3(7):1397–408. doi: 10.1158/2767-9764.CRC-23-0210 (PMC10389052; doi:10.1158/2767-9764.CRC-23-0210)
Supplement: Suppl Figure S11 — Figure S11 shows the effect of CAY10678 on the production of arachidonic acid metabolites from murine BrafV600E melanoma cells [file crc-23-0210-s13.pdf]

Supplementary Figure S11.

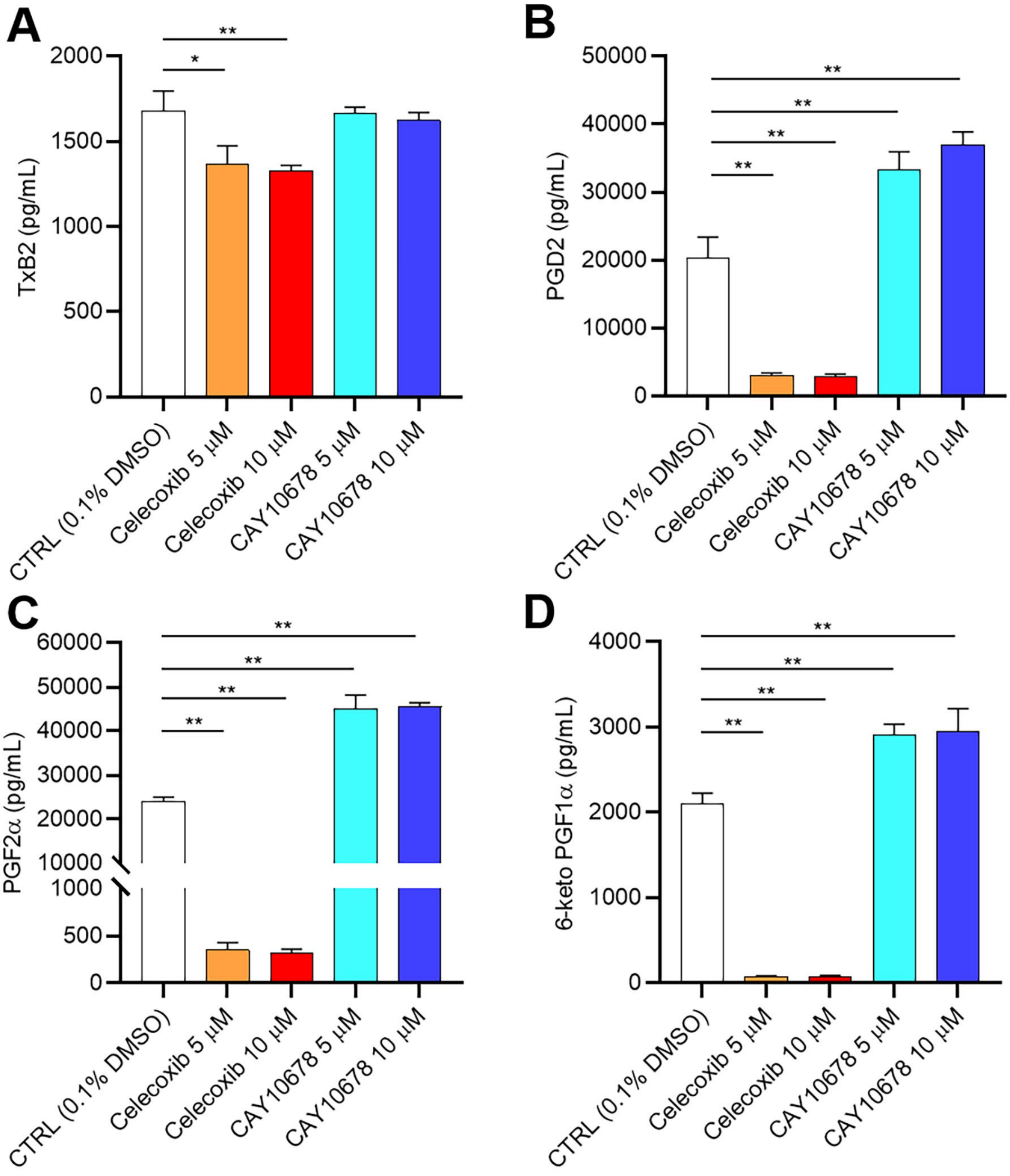

Supplementary Figure S11. The influence of CAY10678 on the production of arachidonic acid metabolites from murine Braf^{V600E} melanoma cells.

A-D, Bar plot showing the concentration of arachidonic acid metabolites released in supernatants in murine Braf^{V600E} melanoma cells treated with different concentrations (0, 5, and 10 μM) of celecoxib and CAY10678. All prostanoids were measured by ELISA (n = 4): TxB2 (A), PGD2 (B), PGF2α (C), and 6-keto PGF1α (D). Graph values represent mean ± SD. Statistical differences between groups were compared using Student's *t*-test. * $p < 0.05$, ** $p < 0.01$.
