# Supplementary material for: Inhibition of Microsomal Prostaglandin E2 Synthase Reduces Collagen Deposition in Melanoma Tumors and May Improve Immunotherapy Efficacy by Reducing T-cell Exhaustion
Source: Cancer Res Commun. 2023 Jul 31;3(7):1397–408. doi: 10.1158/2767-9764.CRC-23-0210 (PMC10389052; doi:10.1158/2767-9764.CRC-23-0210)
Supplement: Suppl Figure S12 — Figure S12 shows the Frequencies of tumor-infiltrating immune cells in tumors treated with celecoxib or CAY10678 as monotherapy or combined with and PD-1 blockade [file crc-23-0210-s14.pdf]

Supplementary Figure S12.

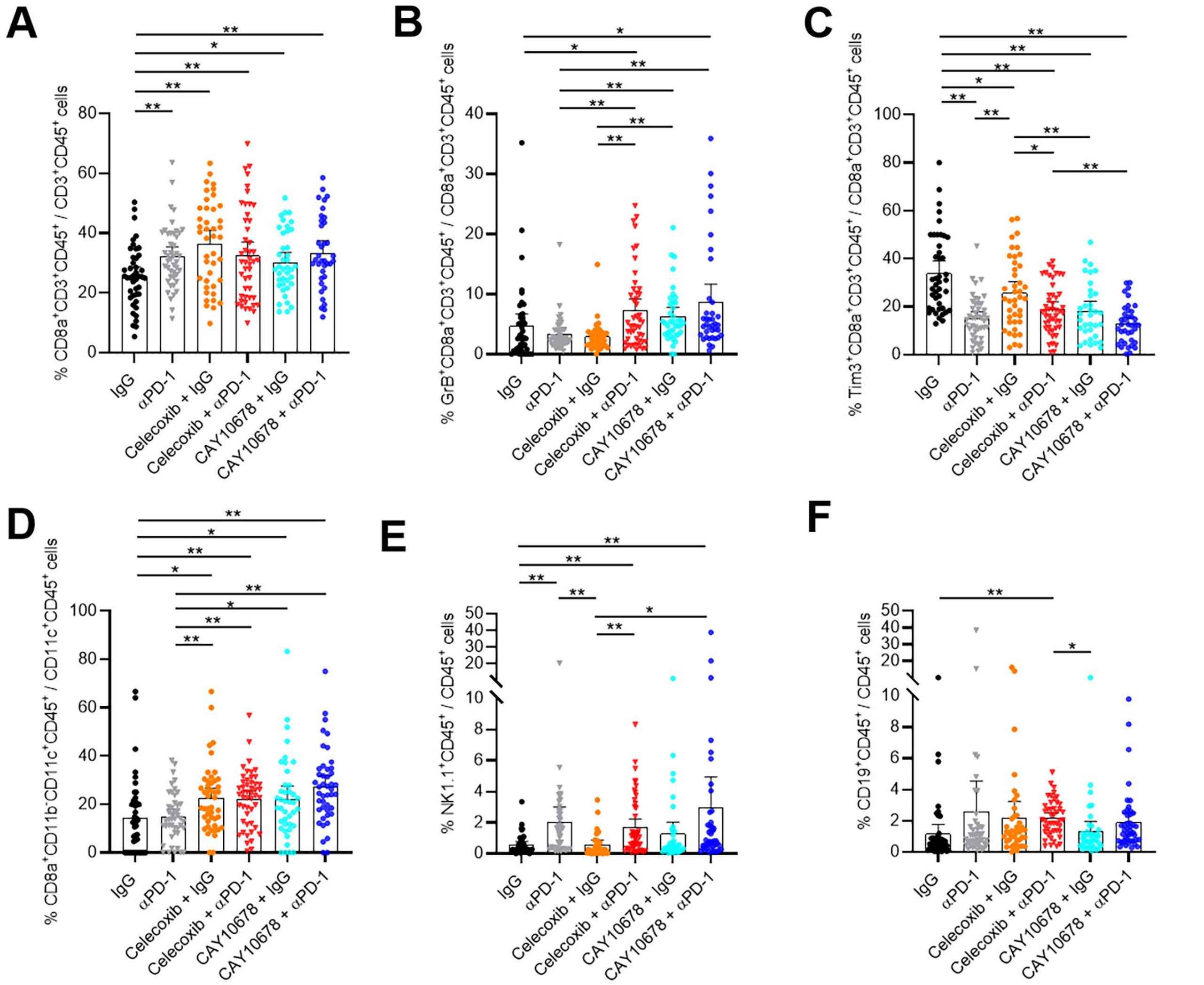

Supplementary Figure S12. Frequencies of tumor-infiltrating immune cells in tumors treated with celecoxib or CAY10678 as monotherapy or combined with and PD-1 blockade.

A-F, Frequencies of tumor-infiltrating immune cells were compared between tumors treated with IgG, αPD-1, celecoxib+IgG, CAY10678+IgG, celecoxib+αPD-1, and CAY10678+αPD-1. The number of tumor-infiltrating immune cells was automatically calculated using Visiopharm software. Shown are the percentage of $CD8a^{+}CD3^{+}CD45^{+}$ cells in total $CD3^{+}CD45^{+}$ cells (A), the percentage of $GrB^{+}CD8a^{+}CD3^{+}CD45^{+}$ cells in total $CD8a^{+}CD3^{+}CD45^{+}$ cells (B), the percentage of $Tim3^{+}CD8a^{+}CD3^{+}CD45^{+}$ cells in total $CD8a^{+}CD3^{+}CD45^{+}$ cells (C), the percentage of $CD8a^{+}CD11b^{-}CD11c^{+}CD45^{+}$ cells in total $CD11c^{+}CD45^{+}$ cells (D), the percentage of $NK1.1^{+}CD45^{+}$ cells in total $CD45^{+}$ cells (E), the percentage of $CD19^{+}CD45^{+}$ cells in total $CD45^{+}$ cells (F). Graph values represent mean ± SD. Significance in difference between two groups was determined by Student *t*-test. *$p < 0.05$, **$p < 0.01$.
